# Unleashing Electrocatalytic Oxygen Evolution Activity: Engineering Spin States in Strained Correlated Oxides for Enhanced Performance

**DOI:** 10.1021/acsnano.5c02188

**Published:** 2025-06-13

**Authors:** Shanquan Chen, Feng-Hui Gong, Xiaowen Li, Yu-Chieh Ku, Cheng-En Liu, Yiyang Nie, Yangyang Si, Shuai Yuan, Jingyu Lu, Hua-Jun Qiu, Kailong Hu, Kaikai Li, Yan Huang, Cheng-Yan Xu, Kelvin Hongliang Zhang, Yun-Long Tang, Lang Chen, Chun-Fu Chang, Zhiwei Hu, Sujit Das, Xiu Liang Ma, Chang-Yang Kuo, Zuhuang Chen

**Affiliations:** † State Key Laboratory of Precision Welding and Joining of Materials and Structures, School of Materials Science and Engineering, 47822 Harbin Institute of Technology, Shenzhen 518055, China; ‡ Shenyang National Laboratory for Materials Science, 71123 Institute of Metal Research, Chinese Academy of Sciences, Shenyang 110016, China; § Department of Physics, 255310 Southern University of Science and Technology, Shenzhen 518055, China; ∥ Department of Electrophysics, 34914 National Yang Ming Chiao Tung University, Hsinchu 30010, Taiwan; ⊥ School of Science, Harbin Institute of Technology, Shenzhen 518055, China; # State Key Laboratory of Physical Chemistry of Solid Surfaces, College of Chemistry and Chemical Engineering, 12466 Xiamen University, Xiamen 361005, China; ∇ 28270 Max-Planck Institute for Chemical Physics of Solids, Dresden 01187, Germany; ○ 29120 Materials Research Centre, Indian Institute of Science, Bangalore, Karnataka 560012, India; ◆ National Synchrotron Radiation Research Center, 101 Hsin-Ann Road, Hsinchu 30076, Taiwan; ¶ Flexible Printed Electronics Technology Center, Harbin Institute of Technology, Shenzhen 518055, China

**Keywords:** LaCoO_3_ thin film, epitaxial strain, spin-state transition, oxygen evolution reaction (OER), OER mechanism

## Abstract

Perovskite oxides have emerged as compelling contenders for catalyzing the oxygen evolution reaction (OER) due to their low cost, high efficiency, and structural flexibility. Nevertheless, unraveling the intricate structure–activity relationships within correlated oxides remains challenging, impeding the rational design of efficient catalysts. Here, using LaCoO_3_ epitaxial thin films as a model system, we illustrate a direct correlation between the spin state and OER activity. Through comprehensive investigations via X-ray absorption spectroscopy, scanning transmission electron microscopy, and first-principles calculations, we pinpoint that the enhanced OER activity observed in the tensile-strained films originates from lattice oxygen oxidation triggered by strain-engineered high-spin Co^3+^. Particularly, the high-spin sites correlated oxygen vacancies during OER lead the reaction into a new pathway, facilitating both the deprotonation of OH* at the metal site and the formation of O–O bonds at the oxygen redox center. Our findings reveal the intricate interplay among strain, spin-state transition, and the transformation of OER mechanism, providing valuable insights for correlated oxide electrocatalysts.

## Graphical abstract

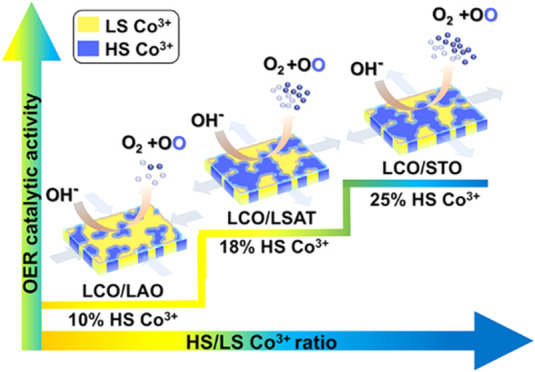

## Introduction

Clean and renewable energy obtained through electrochemical conversion (such as water splitting and fuel cells) is a key to alleviating the energy crisis and environmental issues.^1–4^ Hydrogen gas produced by water electrolysis stands out as a promising clean energy source. Nevertheless, the efficiency of water splitting is hindered by the sluggish kinetics of the oxygen evolution reaction (OER), which can be circumvented to a great extent by the introduction of efficient OER electrocatalysts. Traditional precious metal oxides, including RuO_2_ ^5,6^ and IrO_2_,^7^ have shown excellent OER activity. However, their high prices, limited natural abundances, and stability concerns^3^ pose significant challenges to mass-producing them as durable electrocatalysts. In contrast, low-cost perovskite oxides, characterized as strongly correlated electron systems, exhibit considerable potential in OER electrocatalysis with tunable crystal and electronic structures.^8–11^ Consequently, extensive research efforts have been devoted to exploring perovskite oxide electrocatalysts with excellent OER performance.^12–23^

The adsorbate evolution mechanism (AEM) is the prevailing OER mechanism, which considers only the redox activity of the transition-metal sites, and the lattice oxygen oxidation mechanism (LOM) in OER has received widespread attention in recent years.^24,25^ The AEM involves the transformation of a series of reaction intermediates (OH*, O*, and OOH*) adsorbed on metal active centers at surface, and it is considered as a pH-independent mechanism.^26^ In contrast, the LOM involves the direct participation of oxygen anions from the lattice of catalyst oxides as an active intermediate in the oxygen evolution, and it is considered to have pH-dependent activity. In addition, the LOM challenges the traditional view that OER is a surface reaction.^26^ The key advantage of a LOM-based OER lies in its ability to directly form an O–O bond by bypassing the rate-determining step of AEM (*i.e*. the formation of OOH*).^14,21,24–29^ This renders the LOM-based electrocatalyst usually more effective to enhance the OER activity.^25^ Therefore, an increasing number of researchers have delved into the nature of LOM and endeavored to optimize electrocatalytic performance.^14,26,30–32^ However, a comprehensive understanding of the nature of LOM, such as the electronic structure during the OER process, is still lacking. So far, most investigations have been based on polycrystalline powder systems, making the elucidation of the exact structure–activity relationship for heterogeneous electrocatalysts quite challenging. In order to circumvent multiple variations between different transition-metal elements, it is particularly important to probe the correlation between the electronic structure and catalytic activity in a single material with controlled parameters (e.g., crystal orientation, thickness, and surface roughness). Perovskite cobaltite LaCoO_3_ (LCO) is a classic strongly correlated oxide and has been considered as an efficient electrocatalysts because of its high OER activity and durability.^3,33–36^ The Co^3+^ undergoes a spin-state transition when subjected to external disturbances such as temperature and strain.^33–35,37,38^ In this case, two distinct primary spin states may occur, namely, low spin (LS, *t*_2g_^6^e_g_^0^) and high spin (HS, *t*_2g_^4^e_g_^2^). Different spin states of Co^3+^ can lead to changes in the hybridization of the Co–O bond, thereby altering the OER activity. Although researchers generally understand that the spin state of Co^3+^ has an impact on OER activity,^39–41^ but the direct correlation between the spin state of Co^3+^ and the OER mechanism is still unclear. In consequence, deciphering the elusive impact of the spin states of transition-metal cations on the OER catalysis necessitates a comprehensive understanding of electronic structures, along with electrocatalytic mechanisms. This understanding is crucial for advancing catalyst design and optimizing performance.

In this work, we employ LCO single-crystalline thin films as a model system. Utilizing a diverse set of techniques, including X-ray absorption spectroscopy (XAS) and X-ray linear dichroism spectroscopy (XLD) together with configuration interaction (CI) cluster calculation, aberration-corrected scanning transmission electron microscopy (STEM), and density functional theory (DFT) calculations, we showcase our ability to tune the spin state (HS or LS) and even the OER mechanisms of LCO films under different strains. Noteworthy are the electrochemical measurements, revealing a strain dependence wherein LCO films under tensile strain (with a higher content in HS Co^3+^) exhibit superior OER performance (Figure 1). A pivotal aspect of our investigation indicates that, in comparison to LS Co^3+^ coordinated oxygen, HS Co^3+^ coordinated oxygen is more inclined to facile ligand dissociation, leading to the formation of oxygen vacancies during the OER. Consequently, HS Co^3+^ is more readily reduced to Co^2+^, resulting in the weakening of the Co–O bond. This cascade facilitates the involvement of lattice oxygen as a redox site, further enhancing the OER activity. As evidence, with an increase in the proportion of HS Co^3+^ under tensile strain, the OER activity exhibits significant pH dependence. These findings underscore the close correlation between the spin state and the OER mechanism of LCO, emphasizing the crucial role of the high spin state in activating the oxidation of lattice oxygen, thereby enhancing the OER activity.

**Figure 1:**
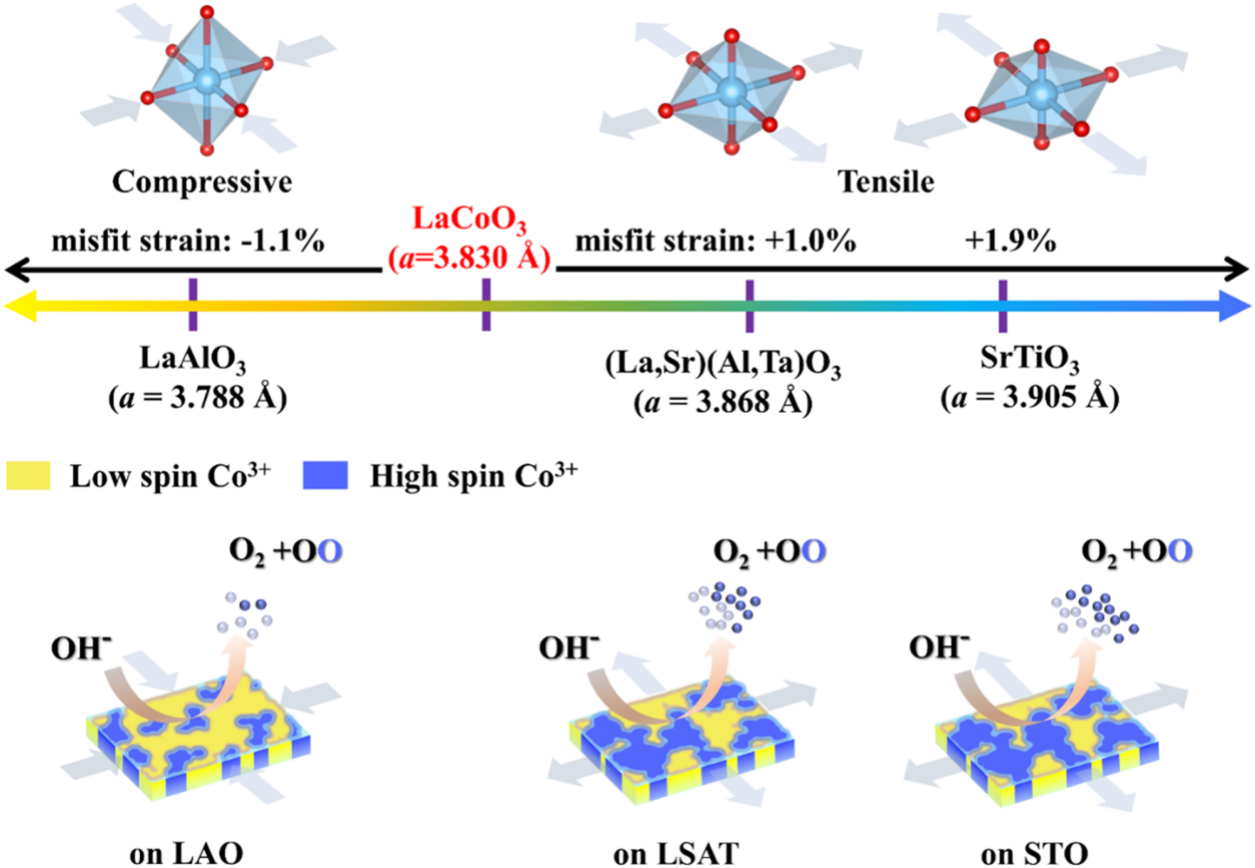
Correlation among strain, spin state, and OER activity in LaCoO_3_ (LCO) films. When the LaCoO_3_ thin film is grown on the LaAlO_3_ substrate, the film is under in-plane compressive strain, causing the out-of-plane lattice to expand; conversely, when LaCoO_3_ thin films are grown on (La, Sr)­(Al, Ta)­O_3_ and SrTiO_3_ substrates with lattice constants larger than it, they will be subjected to in-plane tensile strain, leading to a contraction of the out-of-plane lattice. The spin state of Co^3+^ in LaCoO_3_ thin films can be modulated by controlling the strain. Tensile strain can produce more high-spin Co^3+^ compared to compressive strain, thereby triggering lattice oxygen oxidation to promote the activity of electrocatalytic oxygen evolution reaction.

## Results and Discussion

### Film Growth and Structure Characterization

LCO films (10 nm thick) were grown on La_0.67_Sr_0.33_MnO_3_ (LSMO)-buffered (001)-oriented SrTiO_3_ (STO), (LaAlO_3_)_0.3_(Sr_2_TaAlO_6_)_0.7_ (LSAT), and LaAlO_3_ (LAO) single crystal substrates by pulsed laser deposition. The X-ray diffraction (XRD) 2θ-ω scan reveals that all films are epitaxial and single phase without any impurities (Figure S1). Reciprocal space mapping (RSM) studies reveal that the LCO films are coherently strained to the substrates (Figure S1c-h). The out-of-plane lattice parameters of the LCO films shrink (or expand) compared to the bulk for films grown on tensile-STO and LSAT (or compressive-LAO) substrates because of the Poisson effect. The XRD φ scan further confirms that all LCO films are epitaxially grown on the substrates (Figure S2). The surface morphology of pristine LCO films was determined by an atomic force microscope, exhibiting a smooth film surface with a root-mean-square roughness of ∼150 pm (Figure S3). In addition, we also obtained a high-quality unstrained LCO film (not shown here) on a (001)-oriented YAlO_3_ substrate for strain modulation reference. These high-quality single crystal LCO films eliminate the influence of different structural phases, crystal orientations, and surface roughness on the electrocatalytic activity. This allows us to focus on exploring the relationship between the intrinsic electronic structure of LCO films and the OER activity.

### Electrocatalytic Performance

The OER performance of LCO films was measured using the linear-scan voltammetry (LSV) method in an O_2_-saturated KOH solution (Figure S4). The OER activity of LCO/STO and LCO/LSAT films is noticeably higher than those of LCO/LAO and unstrained LCO films (Figure 2a). The reaction kinetics, as reflected by the Tafel slope, exhibit the same trend (Figure 2b). The current density at a specific voltage (1.73 V vs RHE) and Tafel slope show the higher activity and faster reaction kinetics of LCO films under tensile strain compared to those under compressive strain and unstrained (Figure 2c). The OER reaction kinetics of LCO films can be further confirmed through electrochemical impedance spectroscopy (EIS) measurements (Figure S5a). The Nyquist plots reveal that the charge transfer resistance follows the order LCO/STO < LCO/LSAT < LCO/LAO. These results suggest that the ion transport rate at the electrode–electrolyte interface is significantly higher for LCO/STO thin films compared to LCO/LSAT and LCO/LAO thin films during the OER process, indicating enhanced electrochemical activity in the LCO/STO system. Additionally, the tensile-strained LCO films exhibit pH-dependent OER activity, with the activity increasing more significantly at higher pH (more alkaline) for films under tensile strain (Figure 2d,e); while the compressive-strained LCO films are analogous to the previously reported bulk LCO, which is mainly dominated by AEM and a much weaker pH-dependent behavior is observed.^26^ Moreover, the scan numbers dependent LSV curves reveal a wake-up effect in the OER activity of the tensile-strained LCO films than those under compressive strain, progressively improving with increased scan numbers until it reaches a stable activity (Figure 2g–i and Figure S5b–d), a phenomenon absent in bulk LCO.^36^

**Figure 2:**
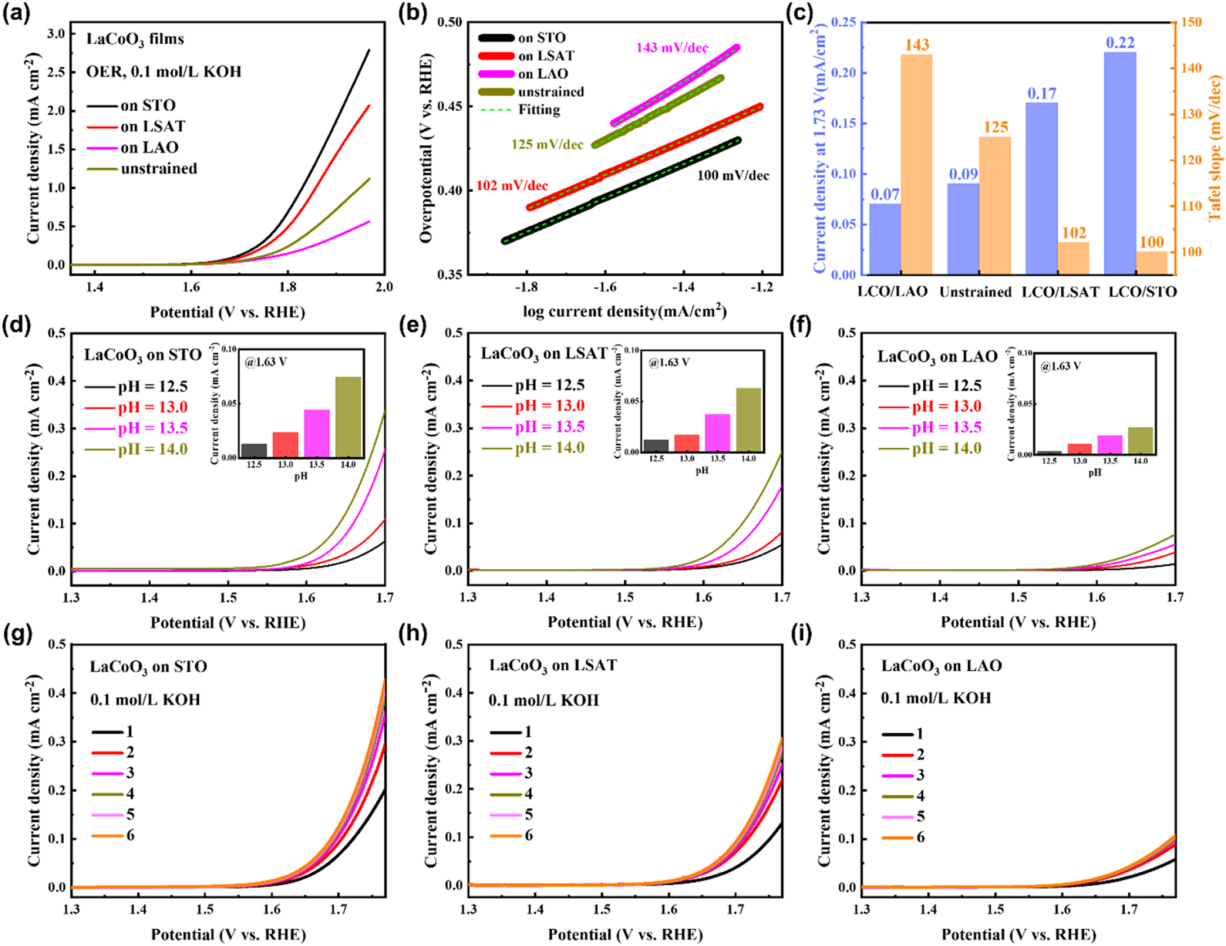
Electrochemical performance of the LCO film. (a) OER linear sweep voltammetry (LSV) polarization curves of LCO films scanned at a rate of 5 mV/s in 0.1 mol/L KOH solution. (b) Tafel plots and fitting curves for LCO films. (c) Comparison of the current density at 1.73 V vs RHE and Tafel slope for LCO films. (d-f) LSV curves of LCO films measured at a rate of 5 mV/s in KOH solution with pH = 12.5–14.0. Inset: pH-dependent the current density at 1.63 V vs RHE. (g-i) Scan numbers dependent OER LSV polarization curves of LCO films scanned at 5 mV/s in 0.1 mol/L KOH. The OER activity of all LCO films exhibited a wake-up effect from the second scan.

To elucidate the mechanism behind the enhanced OER activity of LCO films under a tensile strain, it is necessary to estimate the electrochemically active surface area (ECSA) through electrochemical double-layer capacitance (*C*_dl_) (Figure S6a–e). The *C*_dl_ values for LCO/STO, LCO/LSAT, unstrained LCO, and LCO/LAO are measured to be 106, 87, 76, and 71 μF/cm^2^, respectively. Due to the atomically smooth surface of all LCO films, the contribution of roughness to *C*_dl_ can be ruled out. The ECSA of LCO/STO, LCO/LSAT, unstrained LCO, and LCO/LAO films can be calculated as 1.767, 1.450, 1.270, and 1.187 cm^2^ per unit geometric area, respectively, based on the specific capacitance (*C*_S_ = 60 μF/cm^2^) of the ideal flat oxide surface (inset of Figure S6e). Therefore, the active sites of Co ions have different intrinsic activities during the OER process (Figure S6f), indicating that the LCO films may contain Co ions with different electronic structures, which can be characterized through XPS and XAS.

### Electronic Structure Analysis

To gain insight into the electronic structure of Co ions in LCO films, all samples were measured by XAS at the Co *L*_2,3_ edges (Figure 3a). The Co *L*-edge XAS spectra probe the excitations of electrons from the Co 2*p* core levels to the Co 3*d* unoccupied states. Figure 3a compares the Co *L*_2,3_ XAS spectra acquired from LCO films grown on STO, LSAT, and LAO at 300 K. Consistent with XPS results, the *L*_3_ absorption peak position of all LCO films does not show an evident shift, indicating a consistent Co^3+^ valence state. Additionally, differences in the shape of the Co *L*-edge XAS spectra are observed among LCO films. The spectral features of the Co *L* edge XAS spectra are determined by the multiplet structure caused by the Co 3*d*-3*d* and 2*p*-3*d* Coulomb and exchange interactions, as well as by the local crystal fields and the hybridization with the O 2*p* ligands, making it highly sensitive to changes in the spin state of cobalt ions.^33^ The XAS shape difference (Figure 3a) for LCO films under different strain is similar to previous results on bulk LCO measured at low and high temperatures,^33^ respectively. These results indicate a spin-state variation of Co ions for LCO films, consistent with the results inferred by XPS presented above. By comparing to Sr_2_CoO_3_Cl with pure HS Co^3+^ and EuCoO_3_ with pure LS Co^3+^, Co^3+^ in LCO films exhibit the mixed spin states.

**Figure 3:**
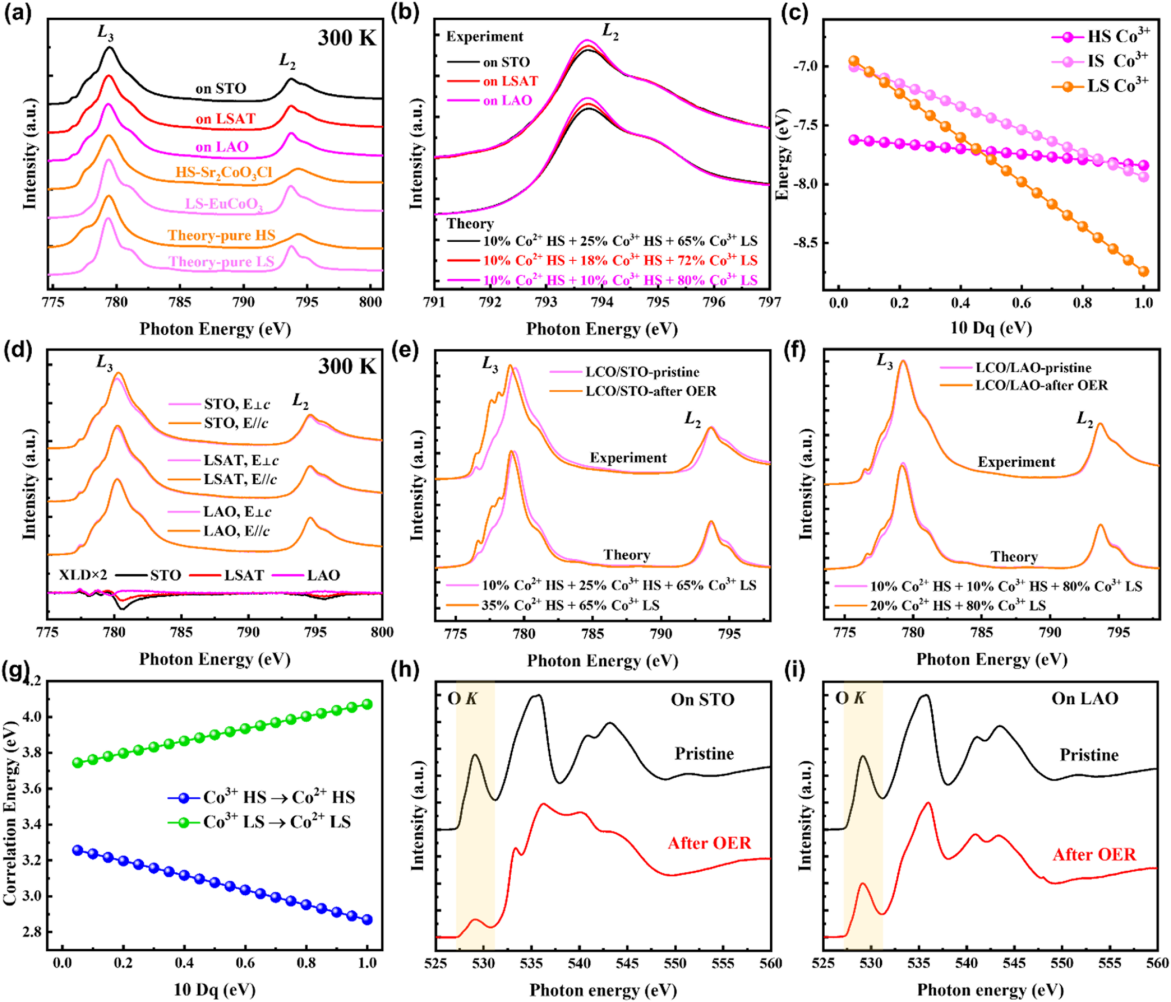
XAS analysis and the CI cluster calculation. (a) XAS spectra of LCO films grown on different substrates at 300 K, including representative reference and theoretical spectra for HS and LS Co^3+^. (b) XAS isotropy spectra of LCO films, reference spectra, and corresponding theoretical fitting spectra with different HS/LS Co^3+^ ratios. (c) Energy level diagram as a function of 10 Dq (which define the e_g_-t_2g_ energy splitting) calculated by CI cluster calculation. (d) Experimental Co *L*_2,3_ XAS spectra and XLD at 300 K for LCO films. (e–f) Experimental Co *L*_2,3_ XAS spectra at 300 K for LCO films grown on STO, LAO before and after OER, along with the corresponding theoretical XAS spectra calculated in the LS-HS scenario. (g) Correlation energy level diagram of HS Co^3+^ → HS Co^2+^ and LS Co^3+^ → LS Co^2+^ as a function of 10 Dq. (h–i) Experimental O *K* edge XAS spectra at 300 K for LCO films grown on STO and LAO before and after OER, respectively.

To gain further insight into the electronic structures of LCO films, the Co *L*-edge XAS spectra of Co^3+^ ions with different spin states were simulated by CI calculations using full atomic multiplet theory (Figure 3b). In principle, Co^3+^ can have three spin states, i.e., the LS, intermediate spin (IS, *t*_2g_^5^e_g_^1^), and HS (Figure S7a–c). By calculating the energy of different spin states with 10*Dq* (which define the *e*_*g*_-*t*_2*g*_ energy splitting) (Figure 3c), it becomes evident that the IS state never becomes the ground state in energy. Therefore, we rule out the IS as the ground spin configuration for Co^3+^ in the films. The XAS spectra for Co^3+^ ions in pure LS and pure HS states were calculated by CI calculations (Figure S7d). By comparing the shape of spectral lines and peak positions, it can be seen that the calculated XAS spectrum for pure HS and LS states can effectively reproduce the experimental XAS spectrum of Sr_2_CoO_3_Cl (with pure HS state Co^3+^)^42^ and EuCoO_3_ (with pure LS state Co^3+^).^43,44^ Therefore, a combination of HS and LS XAS spectra can fit the experimental XAS spectra of LCO films to obtain the corresponding HS/LS ratios of the LCO films under different strains. Figure 3b shows that the experimental XAS spectra agree well with the theoretical spectra calculated with different HS/LS ratios. Correspondingly, all LCO films are in a mixed spin-state system at room temperature, and as the compressive strain transits to tensile strain, the proportion of HS Co^3+^ in LCO films gradually increases.

To further verify the Co^3+^ spin state in LCO films, XLD studies were performed (Figure S8). In principle, the XLD originates from two contributions, namely the orbital anisotropy and the magnetic contribution. Here, the XLD data were collected at 300 K (far higher than the Curie temperature of ∼ 80 K) without applying the magnetic field (Figure 3d). Since the LCO films do not exhibit long-range magnetic ordering at room temperature, the contribution of magnetism to XLD can be ignored. Therefore, the signal of XLD mainly comes from the contribution of orbital polarization. The XLD signal of Co^3+^ in LCO/STO is the strongest, followed by LCO/LSAT, and the smallest is LCO/LAO, indicating that the orbital polarization increases with increasing tensile strain. The orbital polarization in LCO mainly comes from the contribution of HS Co^3+^ because of the uneven distribution of electrons on the *t*_2g_ orbital;^38^ thus, the results of XLD further prove that the content of HS Co^3+^ under tensile strain is higher than that under compressive strain, aligning with the recent publication.^38^

The in-depth investigation of the electronic structure of LCO films shows that the spin state of Co^3+^ in the films are strongly related to the OER electrocatalytic activity. To clarify the intrinsic mechanism, XAS of LCO films after the OER was further investigated. Figure 3e,f shows the Co *L*_2,3_ edge XAS of LCO/STO and LCO/LAO before and after the OER, respectively. Obviously, the LCO/STO produces a large amount of Co^2+^ after electrocatalytic OER, while the LCO/LAO has only a small amount of Co^2+^. To gain insight into the mechanism behind this valence change, the theoretical XAS spectra of LCO films before and after the OER were obtained by CI calculations (Figure 3e,f). We have calculated the XAS of LCO after the OER by replacing all of the HS Co^3+^ with HS Co^2+^, but leaving the same amount of LS Co^3+^ as in the pristine LCO film. It can be seen that the calculated XAS is in very good agreement with the experimental XAS. To gain insight into the spin-state-related valence change in Co, the correlation energy change for HS Co^3+^ → HS Co^2+^ and LS Co^3+^ → LS Co^2+^ was calculated as the function of 10*Dq* (Figure 3g). It is evident that the energy cost of forming LS Co^2+^ is significantly higher than that of HS Co^2+^ because the forming of LS Co^2+^ directly violates Hund’s rule for the Coulomb exchange energy. Therefore, HS Co^2+^ can be generated only by transferring one electron to HS Co^3+^. The presence of Co^2+^ signifies the emergence of oxygen vacancies. The *K* edge XAS (Figure 3h,i) offers additional insights into the created oxygen vacancies. It is evident that the peak at 529 eV of LCO/STO is considerably weaker than that of LCO/LAO after the OER, indicating a higher production of oxygen vacancies in the LCO/STO. This observation further suggests that the increase in oxygen vacancies in the LCO film correlates with HS Co^3+^, and the appearance of HS Co^2+^ is primarily attributed to the loss of lattice oxygen in LCO during the OER. Therefore, the spin-state-associated oxygen vacancies play a crucial role in enhancing the OER activity, suggesting a positive correlation between the spin state of Co ions and the OER performance. The relationship among the proportion of Co^3+^ in the HS state, the generation of oxygen vacancies during the OER process, and the resulting OER activity is summarized in Table S1. It is worth noting that the probing depth of the XAS measurement is approximately 10 nm. The notable alteration in cobalt valence electronic states observed in the XAS results strongly implies that the OER occurred not only on the surface layer of the LCO film but also penetrated into the inner layer of the LCO film. This robustly implies the potential involvement of the LOM in the OER process.

### Aberration-Corrected STEM Analysis

To visually elucidate the changes in crystal and electronic structure before and after the OER, we performed the HAADF-STEM observation of the LCO/STO film (Figures 4 and S9). The pristine LCO exhibits a uniformity of the film, whereas the LCO after the OER showed some dark stripes (Figure 4a,b). Additionally, the LCO after the OER exhibits a larger La–La distance within the dark stripes than the pristine LCO. Mapping of the in-plane and out-of-plane La–La distance revealed that the out-of-plane lattice parameter of the LCO film before and after the OER did not change significantly, However, the in-plane lattice parameter within the dark stripes of the LCO film after the OER increased significantly compared to other regions and the pristine LCO (Figure 4c). To identify the origin of the dark stripes, the integrated differential phase contrast (IDPC) and differential phase contrast (DPC) images corresponding to HAADF-STEM for LCO films before and after OER were carried out (Figure 4d–g). The IDPC images effectively highlight the presence of oxygen atom in the film,^45^ It is evident that the pristine LCO exhibits an intact lattice and oxygen stoichiometry (Figure 4d), whereas the dark stripe in the LCO lattice after OER reveals some oxygen defects (Figure 4e). Furthermore, DPC technology enables the imaging of local electromagnetic fields within materials.^45^ The DPC images show that LCO after the OER exhibits a nonuniform distribution of the atomic electric field compared to pristine LCO (Figure 4f,g). Furthermore, Co *L* edge and O *K* edge electron-energy-loss spectroscopy (EELS) further indicates the presence of oxygen vacancies in the dark stripe regions of the LCO lattice (Figure S10). This observation provides direct evidence of the presence of oxygen vacancies, aligning with the results from XAS, and suggests the potential involvement of LOM during the OER.

**Figure 4:**
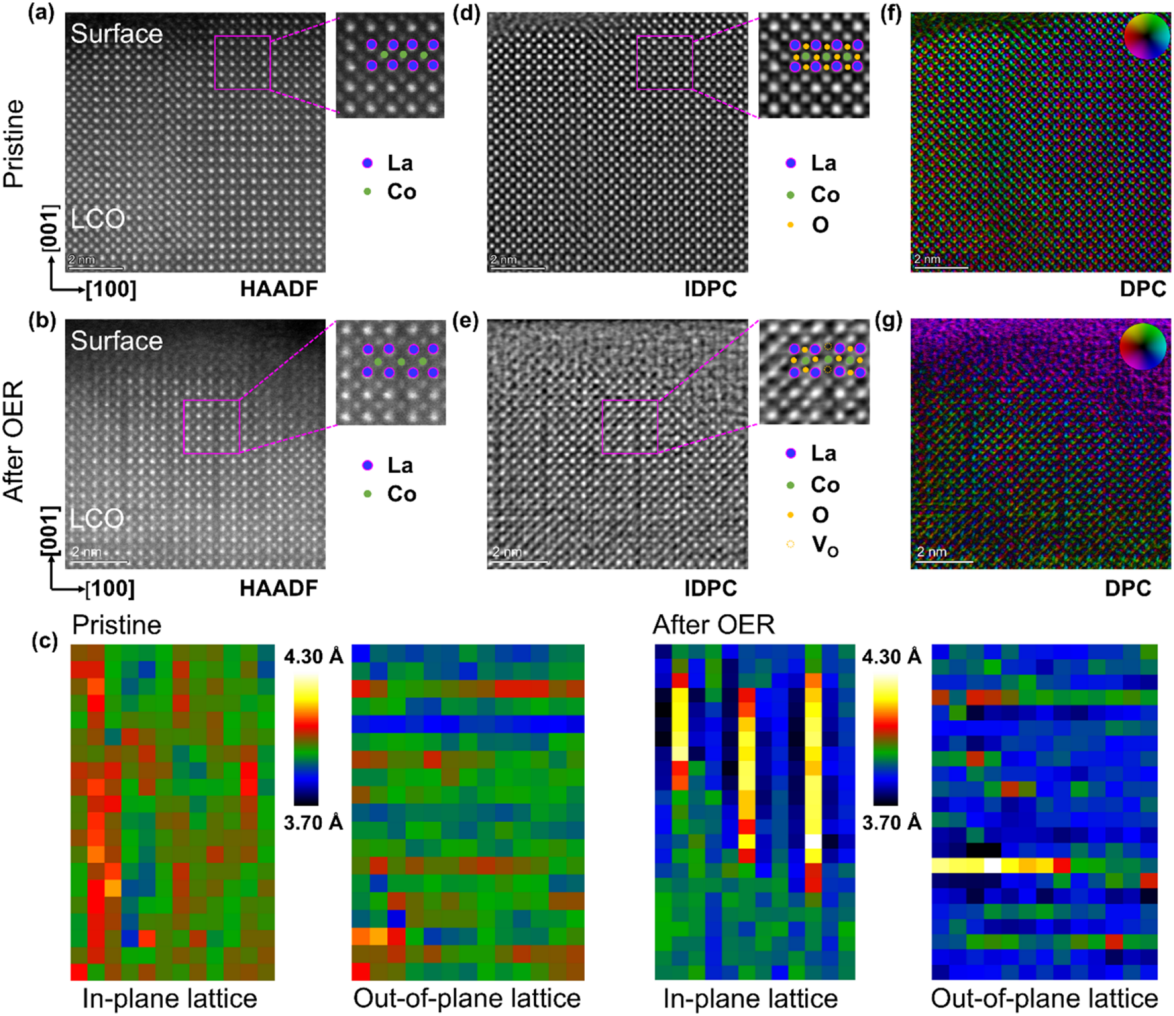
Cross-sectional STEM analysis for LCO films before and after OER. (a, b) HAADF-STEM images; (c) in-plane and out-of-plane lattice parameters; (d, e) Iintegrated differential phase contrast (IDPC) images; (f, g) differential phase contrast (DPC) images for LCO films before and after OER.

### DFT Calculations and OER Mechanisms

To further unravel the underlying OER mechanisms, DFT calculations were carried out. From the projected density of states (DOS) of Co 3*d* in LCO films (Figure S11), it is evident that the DOS of the *e*_g_ orbital (*d*_*x*–*y*_^22^ and *d*_*z*_^2^) under a tensile strain is higher than that under a compressive strain, indicating that the tensile strain is more favorable for the HS Co^3+^ than the compressive strain. Additionally, the DOS of the *t*_2g_ orbital (*d*_*x*y_ *d*_*yz*_ and *d*_*xz*_) suggests that the tensile strain leads to greater anisotropy of the *t*_2g_ orbital than the compressive strain, implying that the tensile strain favor HS Co^3+^. These calculations for the DOS of Co 3*d* are consistent with the results observed in the aforementioned XAS and XLD experiment. Moreover, the DOS of O 2*p* in LCO films reveal that the tensile strain makes the band of the O 2*p* closer to the Fermi level than the compressive strain (Figure S12), indicating that the O connected with HS Co^3+^ is more readily oxidized. Furthermore, the DOS of the O 2*p* in LaCoO_3‑δ_ with oxygen vacancies significantly increases near the Fermi level compared to LCO (Figure S12b). Consequently, the OER in LCO may be conducted through LOM. Based on the experimental and the theoretical analysis, we hypothesize that the lattice oxygen in the LCO films might escape and produce oxygen gas during the OER. This phenomenon could result from the facile loss of oxygen bonded with HS Co^3+^.^38^ The key factor contributing to this relationship is the lower correlation energy change from HS Co^3+^ to HS Co^2+^ compared to that of LS Co^3+^ to LS Co^2+^. This aligns with previous literature reports indicating that the oxygen vacancy formation energy of HS Co^3+^ is lower than that of LS Co^3+^.^46^ Therefore, we propose a revised OER procedure for the AEM and LOM (Figure 5a). First, the lattice oxygen connected to HS Co^3+^ combines with OH^–^ to form intermediates (OH* and O*), ultimately releasing O_2_ and leaving an oxygen vacancy (Figure 5a left, AEM-like). Correspondingly, LCO was transformed into LaCoO_3‑δ_. Notably, based on DFT calculation, in LaCoO_3‑δ_, the O 2*p* band shifts to the Fermi level, designating both the metal and oxygen as redox centers. Subsequently, it transitions to an LOM-like reaction cycle, further enhancing the OER activity (Figure 5a right). Experimentally, the wake-up effect observed in the scan-time-dependent LSV curves proves the transition from AEM-like to LOM-like reaction (Figures 2g–i and S5b–d). Leveraging the electronic configurations, we calculated the evolution of the Gibbs free energy for the OER (occurring via AEM and LOM-like) in LaCoO_3‑δ_ films. For AEM path of LaCoO_3‑δ_ films (Figure S13), the calculated overpotential tendencies are as follows: (η) 0.73*V*_LCO/STO_ < 0.92*V*_LCO/LSAT_ < 1.12*V*_LCO/LAO_ (Figure 5b–d), which are higher than those for LOM-like (Figure 5e–g) (η, 0.63*V*_LCO/STO_ < 0.74*V*_LCO/LSAT_ < 1.09*V*_LCO/LAO_). Gibbs free energy calculations indicate that the subsequent mechanism of the OER of LaCoO_3‑δ_ is mainly dominated by LOM-like. Besides, although the LOM-like reaction involves the oxidation of lattice oxygen, the calculation of valence electrons number reveals that transition metals also participate in the reaction (Figure S14). Through the above-mentioned analysis, it is not surprising that a higher presence of HS Co^3+^ correlates with better OER activity. It turns out that HS Co^3+^ triggers lattice oxygen oxidation, leading to a decrease in overpotential, promoting electron transfer, and accelerating the OER.

**Figure 5:**
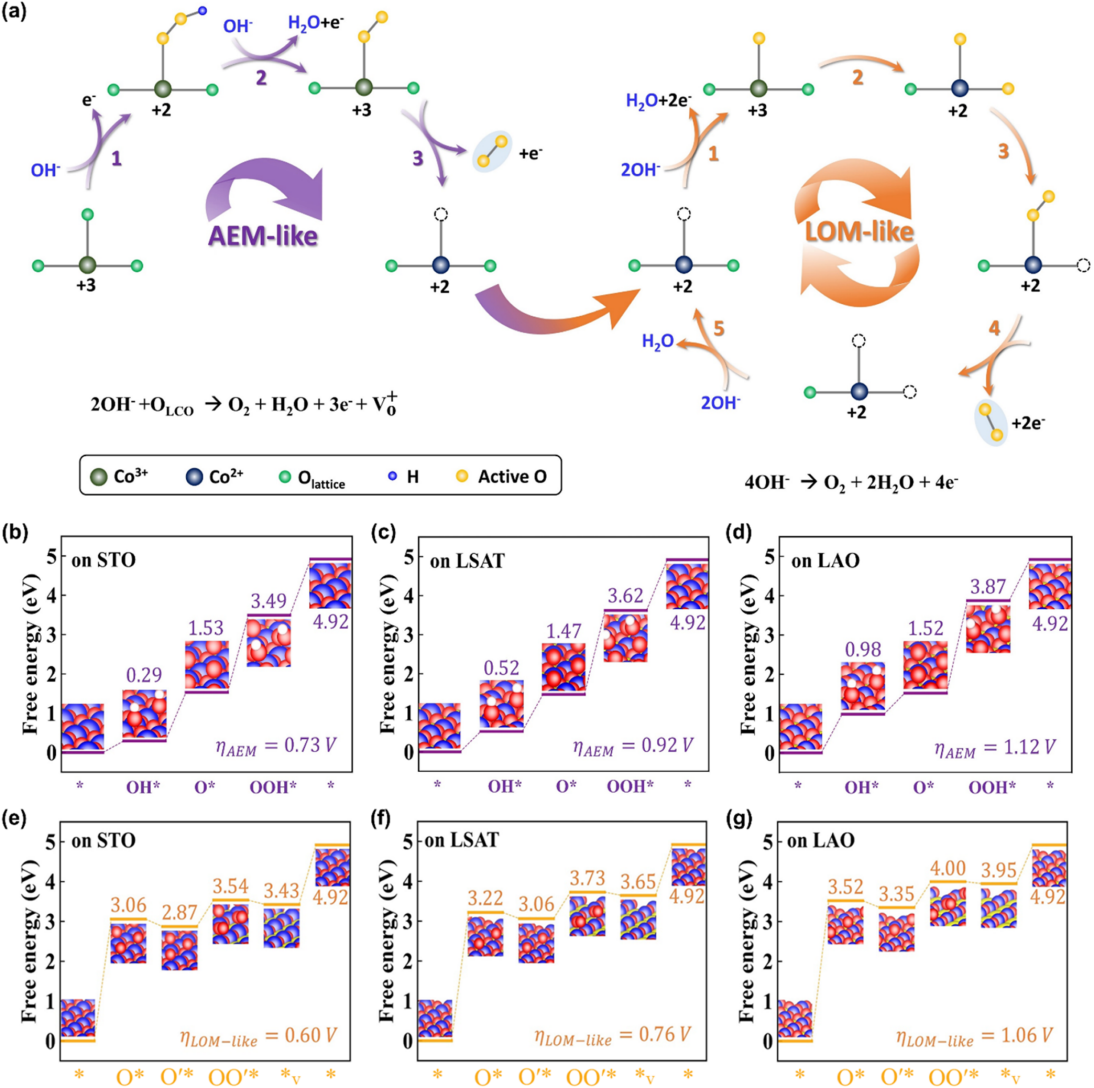
Spin state correlated OER mechanism and Gibbs free energy calculation. (a) OER mechanism for LCO films: HS Co^3+^ dominated the transition from AEM-like to LOM-like mechanism. Simulated Gibbs free energy of LaCoO_3‑δ_ films during OER via (b–d) conventional AEM and (e–g) LOM-like mechanism, respectively. Note: The asterisk “*” represents the active sites in LaCoO_3‑δ_, OH*, O*, and OOH* are oxygen intermediates in the AEM process, while O*, O’*, OO’*, and *v are intermediates in the LOM-like path. For LOM-like, the green asterisk “*” first combines with OH^–^ ions in KOH solution to form O*. This process is mainly dominated by Co metal active sites, causing the valence state of Co ions to rise from +2 to +3. Then, due to electron transfer between Co and O, the valence state of Co ions decreases from +3 to +2, forming the active oxygen species O’*. Then, the two active oxygen species form O–O bonds, producing OO’* intermediates, and releasing oxygen gas to further form active sites *v with more oxygen vacancies. This active site combines with OH- ions in KOH solution and returns to its original * state, forming a LOM-like pathway cycle.

To experimentally confirm the presence of the proposed LOM-like mechanism during the OER on LCO films, we conducted *in situ* differential electrochemical mass spectrometry (DEMS) measurements, a well-proven and effective analytical tool for investigating the OER mechanism^47–49^ (Figure S15). Initially, OER was performed in H_2_^18^O-based 1.0 M KOH solution to obtain the ^18^O-labeled LCO films, Subsequently, we carried out *in situ* DEMS measurement in a H_2_^16^O-based 1.0 M KOH solution. Figure 6 shows the evolution of gaseous products generated during the OER process, providing key insights into the reaction pathway. During the OER process catalyzed by the ^18^O-labeled LCO/STO and LCO/LAO films, both ^32^O_2_ and ^34^O_2_ signals were detected, while no ^36^O_2_ signal was observed. Notably, the ^34^O_2_/^32^O_2_ ratio for LCO/STO (∼0.5%) was higher than that for LCO/LAO (∼0.3%), both exceeding the natural abundance of ^18^O in water (∼0.2%).^26^ Given that all films were thoroughly rinsed with abundant ^16^O water after the ^18^O-labeling process, it is unlikely that the ^18^O species adsorbed on the surface significantly contributed to the observed ^34^O_2_ (^16^O^18^O) signals. The evolution of ^34^O_2_ during the OER process provides compelling evidence supporting our proposed LOM-like OER mechanism. Initially, when conducting the OER with LCO films in an H_2_^18^O-based electrolyte, lattice oxygen involvement in the OER process enables ^18^O from the solution to exchange with lattice oxygen in LCO, allowing ^18^O to enter the lattice, as illustrated in Figure S16a. Subsequently, during the OER of ^18^O-labeled LCO thin films in an H_2_^16^O-based electrolyte, the LOM-like OER pathway leads to the generation of ^34^O_2_, as depicted in Figure S16b. If lattice oxygen is not involved in the OER process of the LCO films, meaning that the OER is governed solely by the adsorbate evolution mechanism (AEM), then after the OER is conducted in H_2_^18^O, the LCO thin films would not incorporate ^18^O into their lattice. As a result, no ^34^O_2_ would be detected when the OER is measured in H_2_^16^O. Therefore, the detection of ^34^O_2_ mass signals in the DEMS results provides direct evidence for the participation of lattice oxygen in the OER process of LCO thin films, particularly when grown on STO under tensile strain.

**Figure 6:**
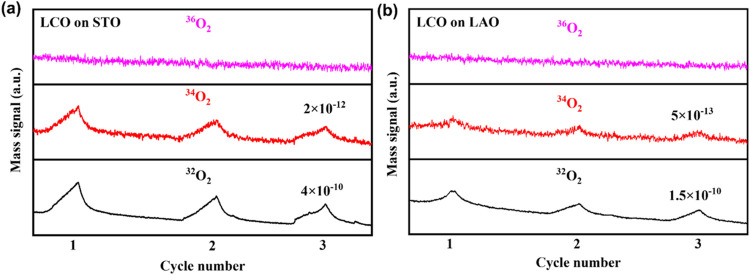
DEMS testing results. The DEMS tracked O_2_ evolution catalyzed by ^18^O-labeled (a) LaCoO_3_/STO and (b) LaCoO_3_/LAO in the H_2_^16^O-based electrolyte during three times of LSV in the potential range of 1.3–2.0 V versus RHE, with a 5 mV s^–1^ scan rate.

## Conclusions

In conclusion, our research demonstrated that epitaxial strain can tune the OER performance and even trigger the lattice-oxygen redox chemistry in correlated oxide LCO. Electrochemical measurements highlight superior OER performance in LCO films under tensile strain, indicative of a Co^3+^ HS state. Importantly, the enhanced OER activity in HS Co^3+^-coordinated oxygen is attributed to a reduction in the formation energy of oxygen vacancies, activating the lattice oxygen oxidation mechanism. Supporting evidence reveals pH-dependent OER activity, particularly pronounced with an increased Co^3+^ HS state in the films. Furthermore, our findings have broader implications for other oxide systems exhibiting spin-state transitions of metal ions, which can be leveraged to enhance OER activity or stability. This includes systems like La_1–*x*_Sr_*x*_CoO_3_,^50,51^ where both Co^3+^ and Co^4+^ have LS, IS, and HS, three different spin states, as well as SrRuO_3_, where Ru^4+^ can transition between LS and HS states.^52^ These insights could guide the design of more efficient and stable OER catalysts through spin-state engineering. These findings underscore the pivotal role of the high spin state in activating lattice oxygen oxidation and establishing criteria for selecting perovskite oxide catalysts based on the intrinsic spin state for effective and cost-efficient OER applications.

## Experimental Methods

### Fabrication of the Epitaxial LaCoO_3_ Single Crystal Films

Ten nm LCO/10 nm LSMO heterostructures were grown via pulsed laser deposition (Arrayed Materials RP-B). The growth of LCO was carried out at an oxygen pressure of 13 Pa at 700 °C with a laser fluence of 1.6 J/cm^2^ and a laser repetition rate of 3 Hz. The growth of LSMO (as a bottom electrode) was accomplished at an oxygen pressure of 20 Pa at 700 °C with a laser fluence of 0.9 J/cm^2^ and a laser repetition rate of 3 Hz. All LaCoO_3_/LSMO heterostructures were cooled down to room temperature (10 °C/min) with 10,000 Pa of oxygen atmosphere after films deposition.

### Characterization of the Crystal and Electronic Structures of the LaCoO_3_ Films

The X-ray 2θ-ω scans and reciprocal space maps were obtained by high-resolution X-ray diffraction (Rigaku, SmartLab 9KW). The surface morphology was determined by an Asylum Research MFP-3D-Infinity AFM. Soft X-ray spectroscopy measurements were carried out in total electron yield mode (TEY) at the TLS11A and TPS45A beamline of the National Synchrotron Radiation Research Center (NSRRC) in Taiwan. The XAS measurements were performed at 300 K using the TEY mode and an X-ray incidence angle of 20° relative to the sample surface. XLD measurements were performed at 300 K and were obtained from the difference of horizontal and vertical polarized light absorption spectra without applied magnetic field under the TEY mode, as shown in Figure S8.

### Configurational Cluster-Interaction (CI) Calculations

LaCoO_3_ is a strongly correlated material, with correlations stemming from both the atomic multiplet effect and the energy cost associated with charge transfer due to covalency. To thoroughly incorporate the correlation effect into our calculations, we utilized conventional CI calculations to determine the correlation energy of the Co ion in LaCoO_3_. This theoretical approach takes into account the complete atomic multiplet effect, configurational interaction, and local solid effect. Three configurations, namely $|3d^{6}\rangle ,|3d^{7}{\mathrm{L̲}}^{1}\rangle$ , and $|3d^{8}{\mathrm{L̲}}^{2}\rangle$ are employed in the calculations for Co^3+^, and the $|3d^{7}\rangle ,|3d^{8}{\mathrm{L̲}}^{1}\rangle$ , and $|3d^{9}{\mathrm{L̲}}^{2}\rangle$ for Co^2+^. The $|{\mathrm{L̲}}^{n}\rangle$ denotes the number of the holes on the ligand orbitals that make the bond with the Co ion. These ligand orbitals are formed by the linear combination of oxygen 2*p* orbitals with respect to the local point symmetry of the Co ion. The energy center of each configuration for Co^3+^ are defined as $|3d^{6}\rangle =0,|3d^{7}{\mathrm{L̲}}^{1}\rangle =\Delta$ , and $|3d^{8}{\mathrm{L̲}}^{2}\rangle =2\Delta +U_{\mathrm{dd}}$ , and for Co^2+^, it is defined as $|3d^{7}\rangle =0,|3d^{8}{\mathrm{L̲}}^{1}\rangle =\Delta +U_{\mathrm{dd}}$ , and $|3d^{9}{\mathrm{L̲}}^{2}\rangle =2\Delta +3U_{\mathrm{dd}}$ . The charge transfer energy Δ = 2.0 eV and the Coulomb repulsion U_*dd*_ = 5.5 eV were used in the calculations.^38^ The hybridization between these three configurations is determined by the parameter of √3*pd*σ which controls the electron hopping between 3*d*_*eg*_ and ${\mathrm{L̲}}_{\mathrm{eg}}$ , and the parameter of 2*pd*π, which controls the electron hopping between 3*d*_t2*g*_ and ${\mathrm{L̲}}_{t2g}$ . We take *pd*σ = 1.7*eV* and *pd*π = 0.74 *eV* for both high spin and low spin simulations.^38^ The electron–electron full-multiplet effect, which is a key determinant of the Co^3+^ spin state, was included in each configuration. The Slater integrals of F_2_(dd) = 12.662­(eV)*SIred, F_4_(dd) = 7.916­(eV)*SIred, which were determined by the Hartree–Fock approximation using the R. D. Cowan code RCN36K, were used in the calculations to account for the atomic full-multiplet effect, where the parameters for Slater integral reductions (SIred = 0.75) were further employed to compromise the solid-state effect. The correlation energy shown in Figure 3c,g represents the ground state energy obtained from CI calculations with the aforementioned parameters.

To simulate LS and HS XAS, three configurations of $|\mathrm{c̲}3d^{7}\rangle ,|\mathrm{c̲}3d^{8}{\mathrm{L̲}}^{1}\rangle$ , and $|\mathrm{c̲}3d^{9}{\mathrm{L̲}}^{2}\rangle$ were future employed, where c̲ denotes a hole that appeared in the Co 2*p* core level after an electron transition to the 3*d* orbital by X-ray. The energy centers of these three configurations are $|\mathrm{c̲}3d^{7}\rangle =\varepsilon_{\mathrm{XAS}},|\mathrm{c̲}3d^{8}{\mathrm{L̲}}^{1}\rangle =\varepsilon_{\mathrm{XAS}}+\Delta +U_{\mathrm{dd}}-U_{\mathrm{dp}}$ , and $|\mathrm{c̲}3d^{9}{\mathrm{L̲}}^{2}\rangle =\varepsilon_{\mathrm{XAS}}+2\Delta +{3U}_{\mathrm{dd}}-2U_{\mathrm{dp}}$ , where the ε_*XAS*_ is the energy required to excite an electron from the Co 2*p* to the Co 3*d* level. The *U*_*dp*_ = 7.0 eV was used and the Slater integrals of F_2_(d-p) = 7.899*SIred, G_1_(d-p) = 5.947*Sired, and G_3_(d-p) = 3.384*SIred (with Sired = 0.75) for the Co 2*p*-3*d* multiplet were further included in the calculation.

### Electrochemical Measurements of LaCoO_3_ Films

Electrochemical measurements were performed in a three-electrode system on an electrochemical workstation (CorrTest, CS350H) using a saturated Ag/AgCl electrode as the reference electrode, a Pt strip as the counter electrode, and a self-assembled thin film electrocatalytic test device with LCO films as the working electrode (Figure S4). High purity Ag wires were affixed to LSMO films (bottom electrode) under LCO films with a silver paint, and then the silver paint, exposed Ag wires, the backs and sides of device were covered with nonconductive, chemically resistant, and catalytically inactive epoxy, in order to make sure only the LCO surface was exposed to the electrolyte. LSV was performed in 0.1 and 1.0 mol/L KOH solution at a scan rate of 5 mV/s. Electrochemical impedance spectroscopy (EIS) was carried out at 1.63 V with an amplitude of 10 mV.

### Aberration-Corrected STEM Characterization

Cross-sectional samples for the STEM observations were prepared by slicing, grinding, dimpling, and finally ion milling by using Gatan Precision Ion Polishing System 695. In the initial stage of ion milling, the voltage was set to 6 kV and the ion beam incident angle was 7°. The voltage and incident angle were gradually reduced to 3.8 kV and 4°, respectively, taking 10 min as a step until the appearance of colored stripes. HAADF-STEM, IDPC, and DPC images were acquired by using a Thermo Scientific Spectra 300 kV microscope with a high-brightness field-emission gun and double aberration correctors. The positions of atom columns in HAADF-STEM images were determined on the basis of the 2D Gaussian fitting, which was carried out by using the MATLAB software. The lattice spacing was deduced.

### DFT Calculations

The first-principles calculations were conducted based on the framework of Density Functional Theory (DFT), leveraging the Vienna ab initio Simulation Package (VASP) code.^53^ We utilized the Strongly Constrained and Appropriately Normed (SCAN) meta-Generalized Gradient Approximation (meta-GGA) as the exchange-correlation functional in our calculations.^54^ A stringent set of convergence criteria was employed to ensure the accuracy of our results. Specifically, an energy tolerance of 10^–5^ eV was adopted, in conjunction with a force tolerance of 0.01 eV/Å, with these calculations performed under a cutoff energy of 500 eV. We employed automatic k-mesh generators with a resolution value (*l*) of 0.03 for the k-points, where *l* denotes the value resolved between adjacent *k*-points in the reciprocal cell and the unit is 2π/Å. The total number (*N*) of *k*-points was determined using the following equation

1 $$N=\max\left(1,\frac{\left|\vec{b}\right|}{l}\right)$$

In this formula, $\vec{b}$ represents the reciprocal lattice vector in a specific direction. This approach ensures that our first-principle calculations are robust, offering reliable insights into the OER process on various surfaces.

### Differential Electrochemical Mass Spectrometry (DEMS) Measurements

DEMS measurements were carried out to clarify the mechanism of the OER of LCO epitaxial films using a QAS 100 device (Linglu Instruments, Shanghai). A saturated Ag/AgCl electrode was used as the reference electrode, and a Pt strip was used as the counter electrode. A self-assembled thin film electrocatalytic test device was used with LCO films as the working electrode. First, the LCO films were labeled with ^18^O isotopes by performing 5 LSV cycles at a scan rate of 5 mV/s in ^18^O-labed 1.0 M KOH solution (the potential window was 1.3–2.0 V vs RHE). Afterward, ^18^O-labeled LCO films were rinsed with abundant deionized-water (H_2_^16^O), dried it with nitrogen gas flow, and then performed the *in situ* DEMS measurement in conventional H_2_^16^O-based 1.0 M KOH solution. The LCO films were carried out in LSV cycles in ^16^O KOH solution at the above potential window and scan rate. Simultaneously, the oxygen gas of different molecular weights generated during OER process were measured in real time by mass spectrometry. Since all films were thoroughly rinsed with abundant ^16^O water after ^18^O-labeling, it is unlikely that ^18^O species adsorbed on the surface contribute substantially to the observed ^34^O_2_ (^16^O^18^O) signals. Therefore, it can be determined to investigate the participation of lattice oxygen from LCO films in the OER by measuring the ^34^O_2_ mass signals.

## Supplementary Material



## Notes

Figures S1–S16, including characterization of structure of LaCoO_3_ films grown on different substrates (S1), the XRD φ scans and the epitaxy of LCO and LSMO thin films (S2), the surface morphology before and after OER (S3), electrocatalytic measurement (S4), electrochemical characterization (S5), active surface area and intrinsic activity (S6), spin state and XAS analysis (S7), XLD analysis (S8), Table S1, the STEM and EDS analysis (S9), the STEM-EELS of LCO films before and after OER (S10), DOS calculations for Co 3*d* in LCO films (S11), DOS calculations for O 2*p* (S12), schematic diagram of AEM for LaCoO_3‑δ_ films (S13), calculation of valence electron number (S14), the schematic diagram of the device for the ^18^O isotope labeling and the DEMS testing system (S15), and the OER pathways on LaCoO_3_ thin films (S16) (PDF)
