# Supplementary material for: Unleashing Electrocatalytic Oxygen Evolution Activity: Engineering Spin States in Strained Correlated Oxides for Enhanced Performance
Source: ACS Nano. 2025 Jun 13;19(26):23647–58. doi: 10.1021/acsnano.5c02188 (PMC12257641; doi:10.1021/acsnano.5c02188)
Supplement: Supplementary file 1 [file nn5c02188_si_001.pdf]

Supplementary materials for

Unleashing electrocatalytic oxygen evolution activity: engineering spin states in strained correlated oxides for enhanced performance

Shanquan Chen,[†,∇] Feng-Hui Gong,[‡,∇] Xiaowen Li,[※,∇] Yu-Chieh Ku,[□] Cheng-En Liu,[□] Yiyang Nie,[†] Yangyang Si,[†] Shuai Yuan,[†] Jingyu Lu,[€] Hua-Jun Qiu,[†] Kailong Hu,[†] Kaikai Li,[†] Yan Huang,[†] Cheng-Yan Xu,[†] Kelvin Hongliang Zhang,[₴] Yun-Long Tang,[‡] Lang Chen,[※] Chun-Fu Chang,[₵] Zhiwei Hu,[₵] Sujit Das,[□] Xiu Liang Ma, [‡,*] Chang-Yang Kuo[□,₺,*] and Zuhuang Chen[†,϶,*]

[†] State Key Laboratory of Advanced Welding and Joining of Materials and Structures, School of Materials Science and Engineering, Harbin Institute of Technology, Shenzhen, 518055, China

[‡] Shenyang National Laboratory for Materials Science, Institute of Metal Research, Chinese Academy of Sciences, Shenyang, 110016, China

[※] Department of Physics, Southern University of Science and Technology, Shenzhen, 518055, China

[□] Department of Electrophysics, National Yang Ming Chiao Tung University, Hsinchu, 30010, Taiwan

[€] School of Science, Harbin Institute of Technology, Shenzhen 518055, China

[₴] State Key Laboratory of Physical Chemistry of Solid Surfaces, College of Chemistry and Chemical Engineering, Xiamen University, Xiamen, 361005, China

[₵] Max-Planck Institute for Chemical Physics of Solids, Dresden, 01187, Germany

[□] Materials Research Centre, Indian Institute of Science, Bangalore, Karnataka 560012, India

[₺] National Synchrotron Radiation Research Center, 101 Hsin-Ann Road, Hsinchu 30076, Taiwan

[϶] Flexible Printed Electronics Technology Center, Harbin Institute of Technology, Shenzhen, 518055, China

[∇] These authors contributed equally to this work.

* Corresponding authors: Ma, X. (xlma@imr.ac.cn), Kuo, C.-Y. (changyangkuo@nycu.edu.tw), Chen, Z. (zuhuang@hit.edu.cn).

This PDF file includes:

Fig. S1 to S16

Table S1

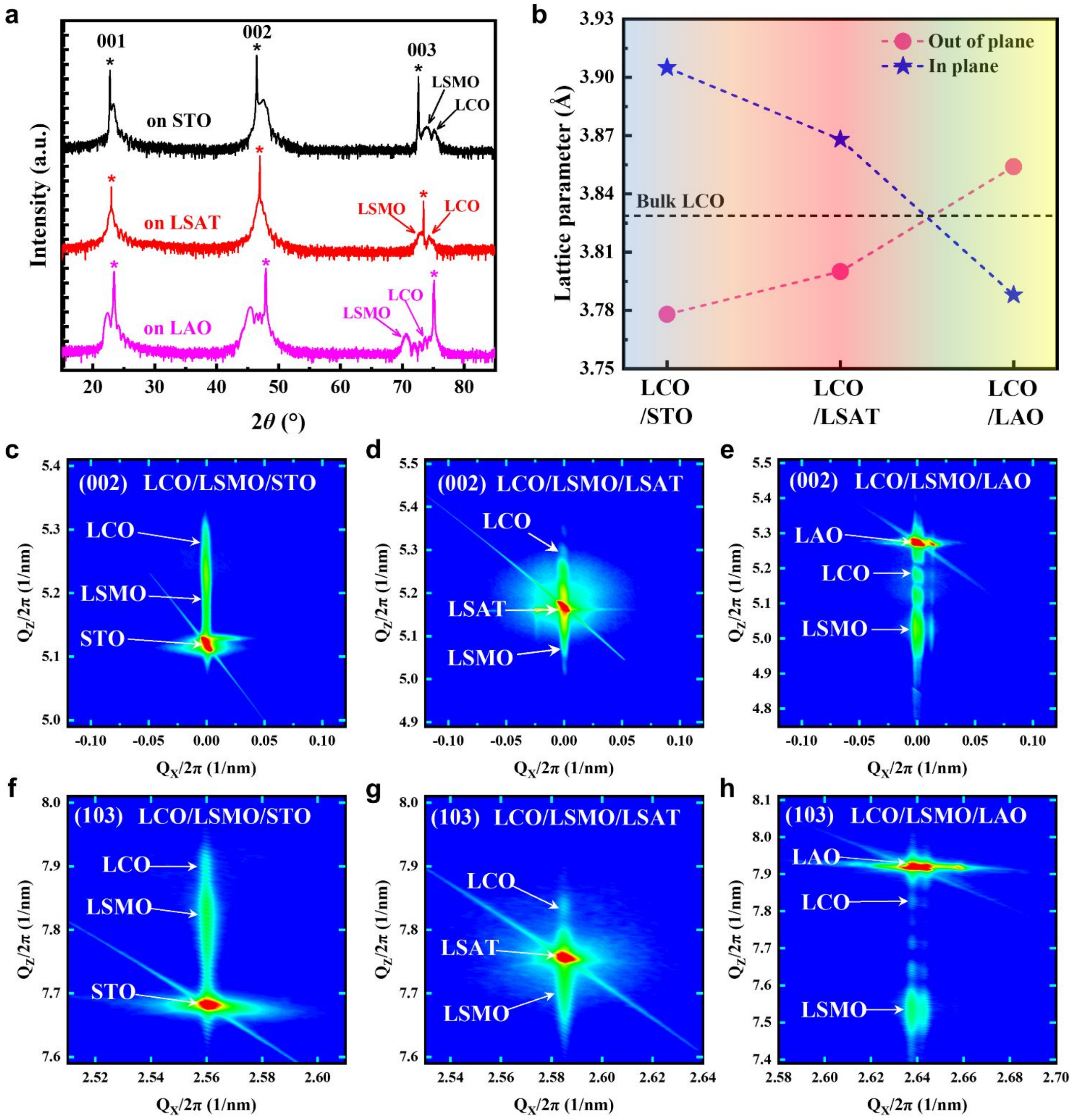

Figure S1 Structure of $LaCoO_3$ films grown on different substrates. (a) X-ray diffraction patterns around the (001), (002) and (003) reflections of LCO films. The peaks of substrates are indicated with an asterisk "*". (b) The out-of-plane and in-plane lattice parameters of LCO films grown on different substrates. Reciprocal space mappings (RSM) of LCO films grown on (c,f) STO, (d,g) LSAT and (e,h) LAO around the substrates' (002) and (103) reflections.

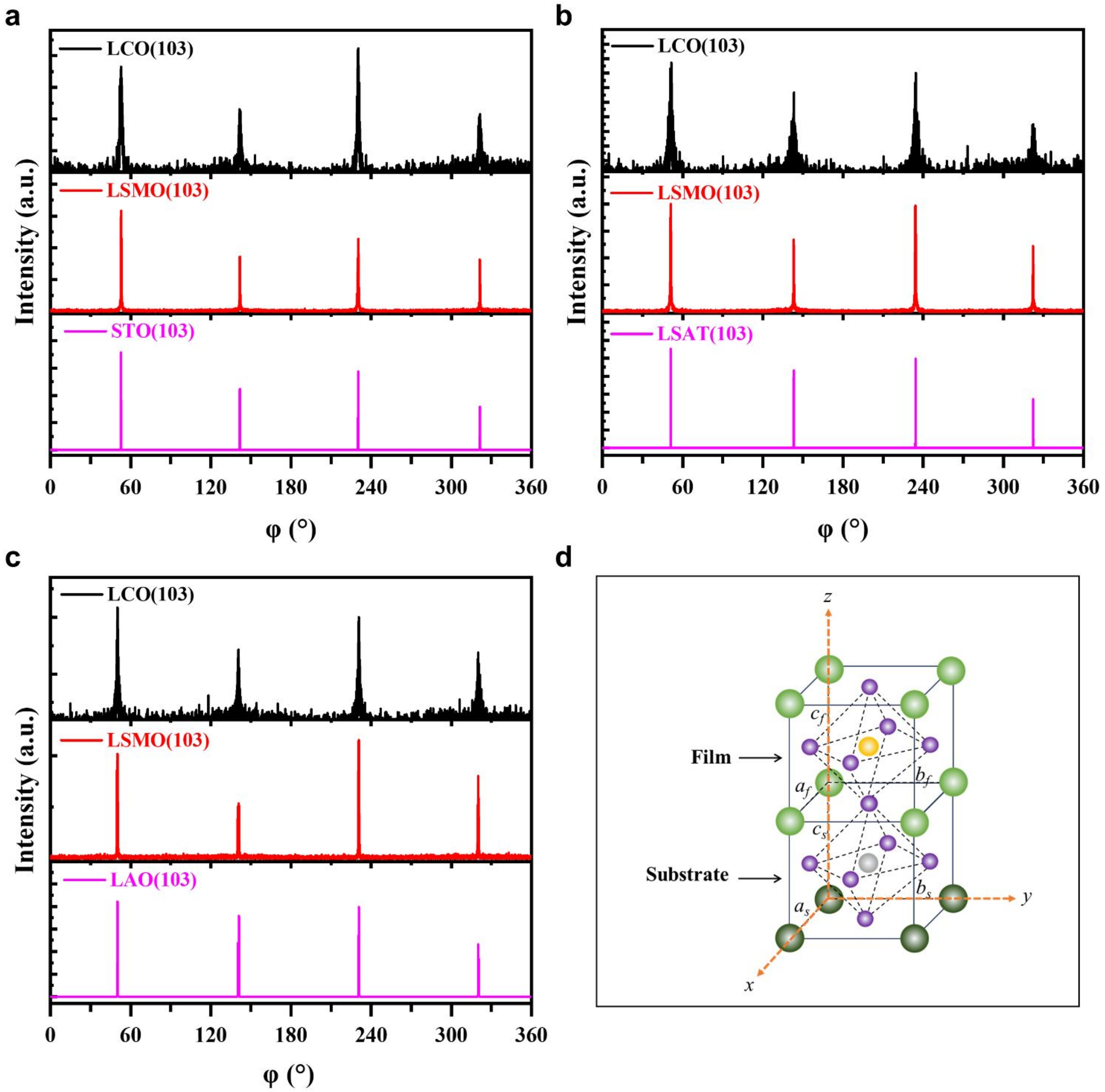

Figure S2 The XRD φ scans and the epitaxy of LCO and LSMO thin films. The XRD φ scans of LCO films grown on (a) STO, (b) LSAT and (c) LAO around the (103) reflection indicate that all LCO films are epitaxially grown on the substrates, as shown in schematic diagram (d).

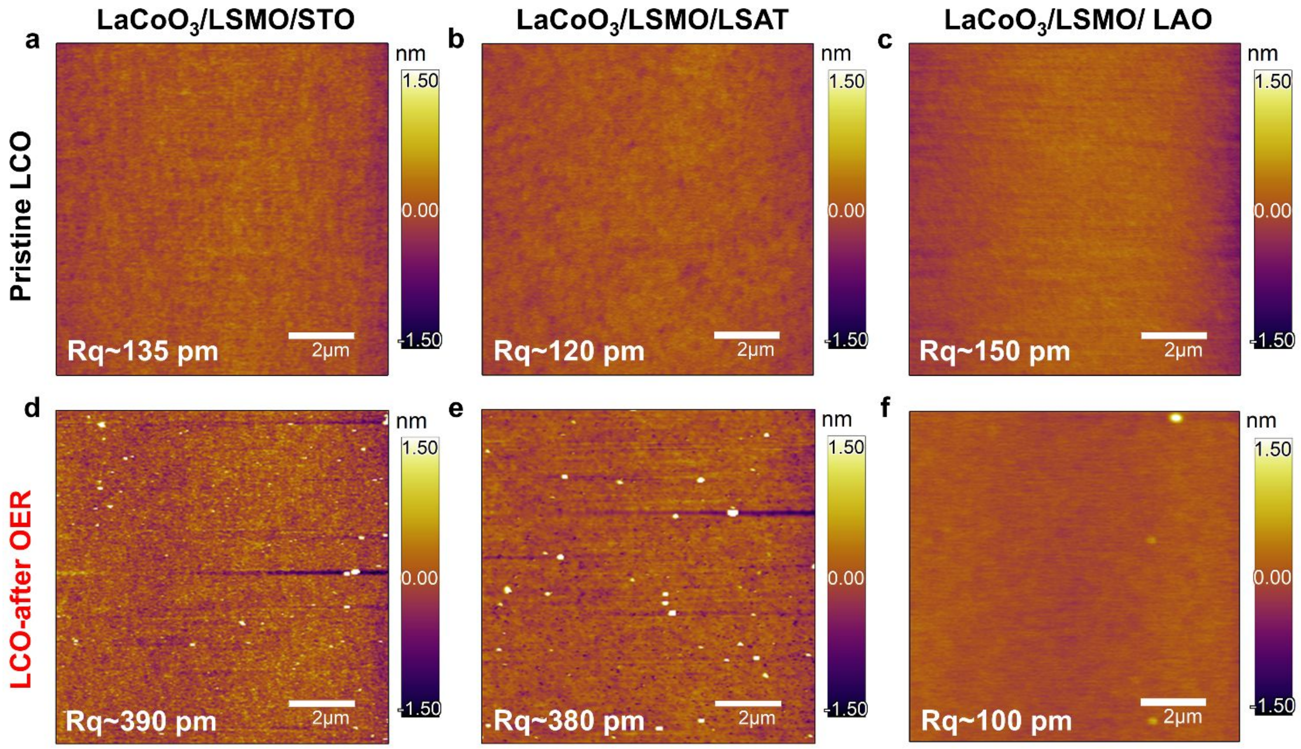

Figure S3 The surface morphology before and after OER. The AFM images of the surface roughness of LCO films grown on (a,d) STO, (b,e) LSAT and (c,f) LAO before and after OER. All LCO films exhibit atomically smooth surface. Compared with the pristine LCO films, the surface of the LCO films grown on STO and LSAT after OER becomes rough, while the surface of the LCO film grown on LAO does not change significantly. The root mean square (RMS) roughness of all LCO films is less than 400 pm.

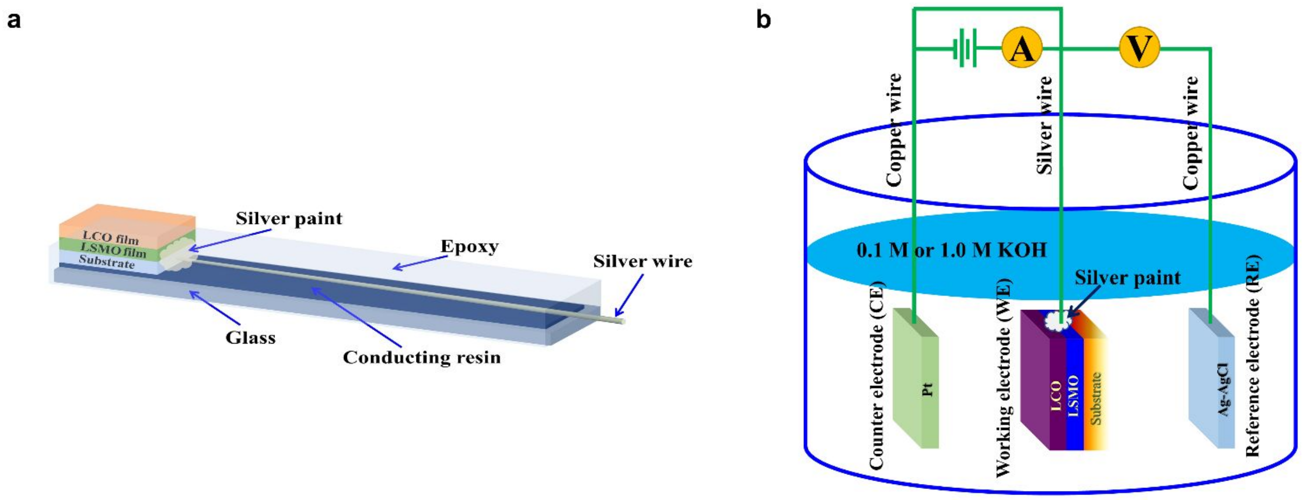

Figure S4 Electrocatalytic measurement. The schematic of (a) device for LCO films with a 10 nm LSMO bottom electrode and (b) OER electrocatalytic measurement in 0.1 M or 1.0 M KOH solution. In this framework, electrochemical measurements were performed using $LaCoO_3$ films as the working electrode, a Pt strip as the counter electrode and a saturated Ag/AgCl electrode as the reference electrode.

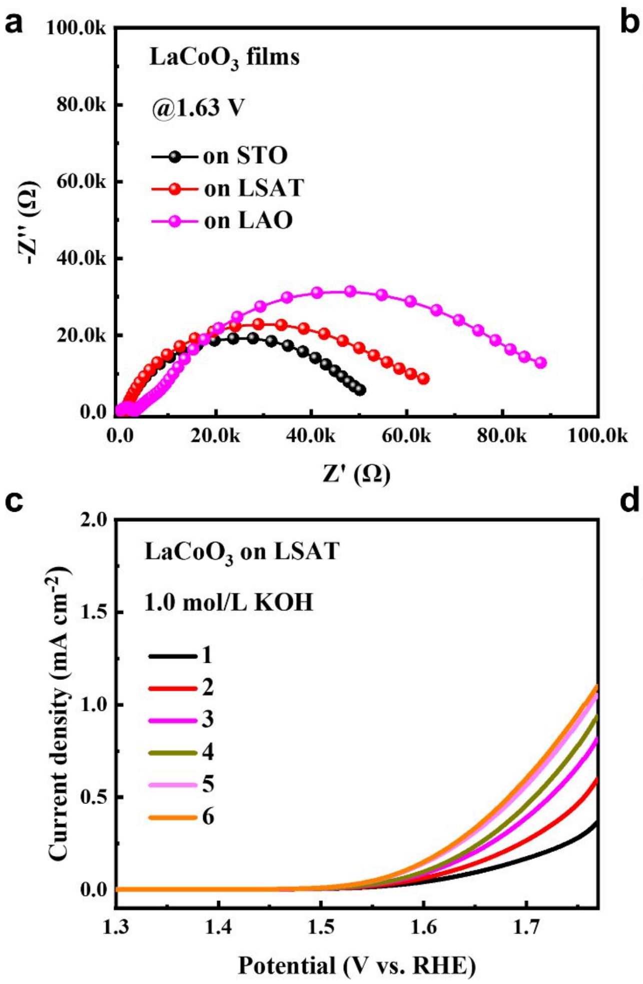

Figure S5 Electrochemical characterization. (a) Nyquist plots for LCO films measured at 1.63 V vs. RHE in 0.1 M KOH aqueous electrolyte. (b-d) The LSV polarization curves of $LaCoO_3$ films scanned at a rate of 5 mV/s in 1.0 mol/L KOH.

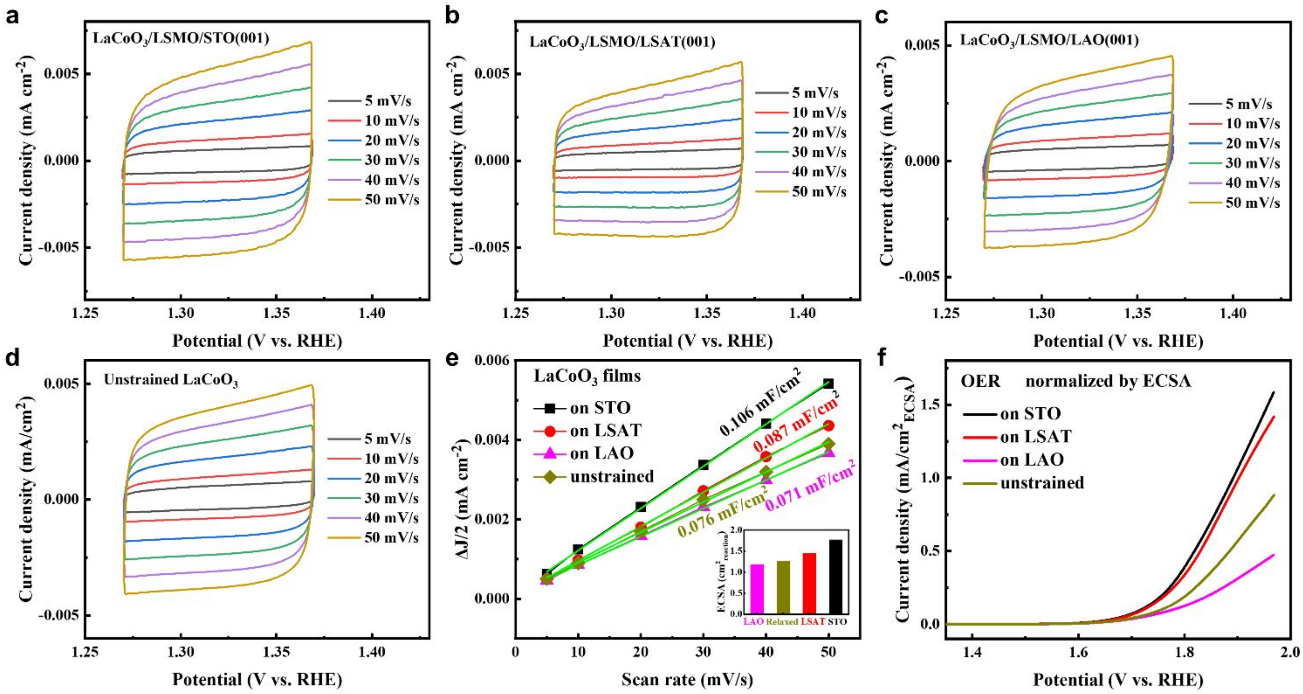

Figure S6 Active surface area and intrinsic activity. (a-d) Electrochemical active surface area (ECSA) analysis through the CV measurement at different scan rates. (e) The plots of the capacitive current at 1.32 V vs RHE as a function of the scan rate of LCO films grown on different substrates. Inset: ECSA obtained from double-layer capacitance. (f) The OER activity normalized by ECSA.

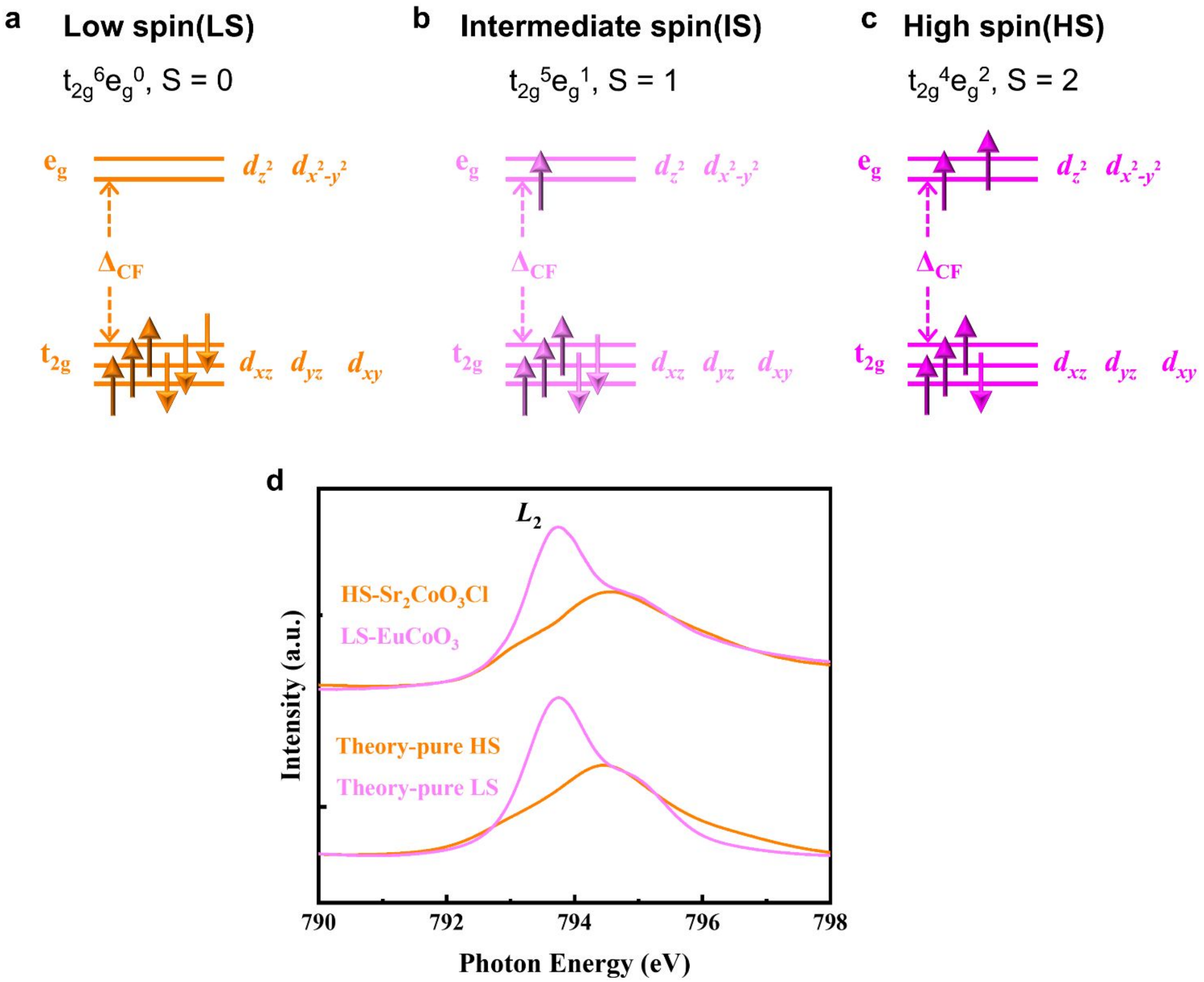

Figure S7 Spin state and XAS analysis. (a-c) The electronic structures of different spin states of Co^{3+} in $LaCoO_3$ films. (d) XAS isotropy spectra for pure HS (Sr_2CoO_3Cl) and LS ($EuCoO_3$) reference spectra and corresponding theoretical fitting spectra by CI calculation.

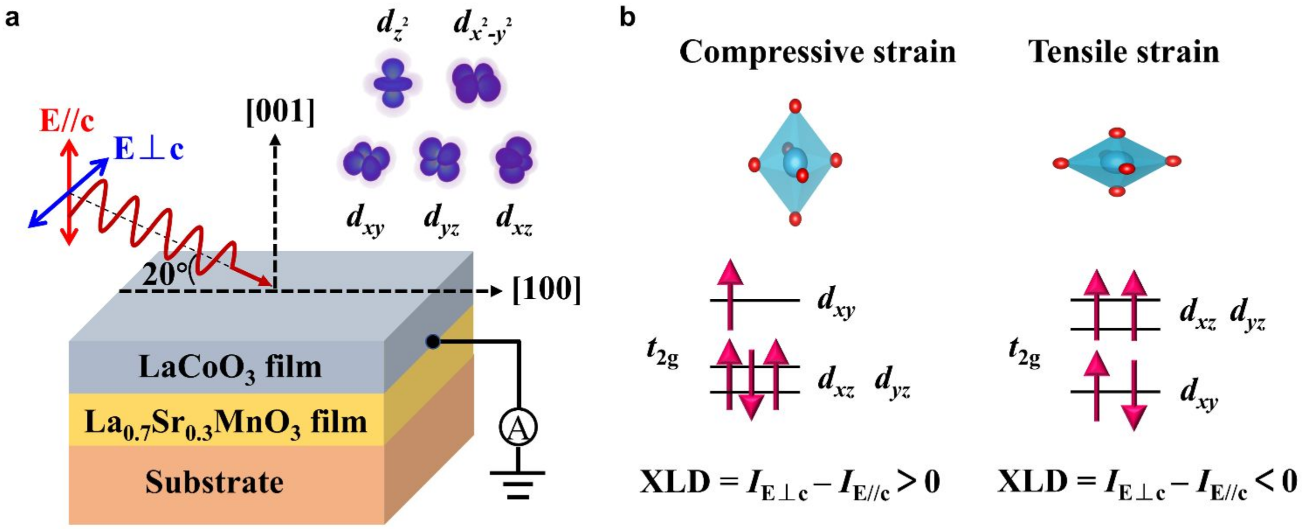

Figure S8 XLD analysis. (a) Schematic diagram of the XLD process; (b) The electron occupation and XLD symbol of t_{2g} orbitals in LCO films under compressive and tensile strains.

Table S1. Strain, oxygen vacancy levels, and OER activity of LCO thin films grown on STO, LSAT, and LAO substrates

	**LCO/LAO**	**LCO/LSAT**	**LCO/STO**
**Strain**	-1.1%	+1.0%	+1.9%
**Co^{3+} HS content** (%)	10%	18%	25%
**Oxygen vacancy level**: δ (pristine)	0.050	0.050	0.050
**Oxygen vacancy level**: δ (after OER)	0.100	0.140	0.175
**Current density at 1.73 V** ($mA\ cm^{-2}$) in 0.1 mol/L KOH	0.07	0.17	0.22

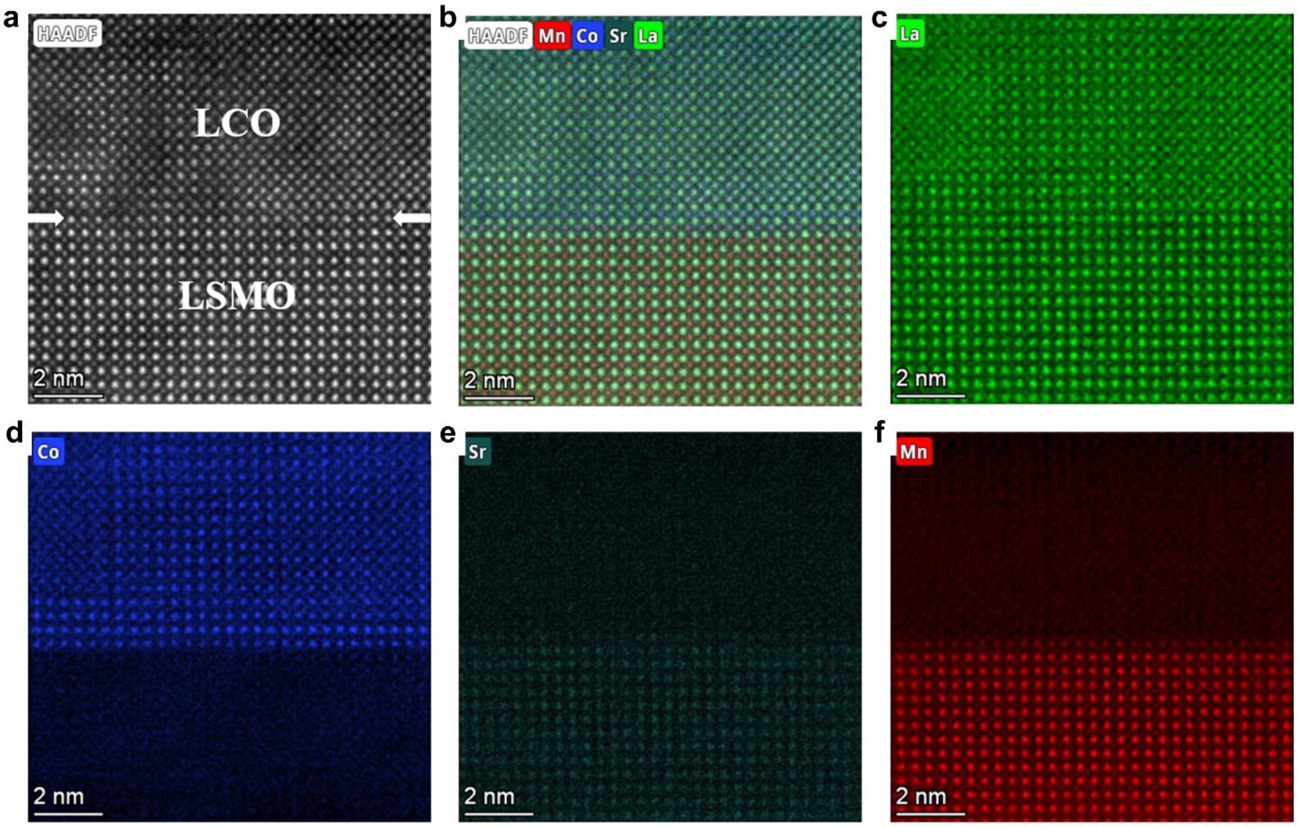

Figure S9 STEM and EDS analysis. (a)The HAADF-STEM images of LCO film and LSMO bottom electrode and corresponding (b-f) the atomic-scale EDS maps.

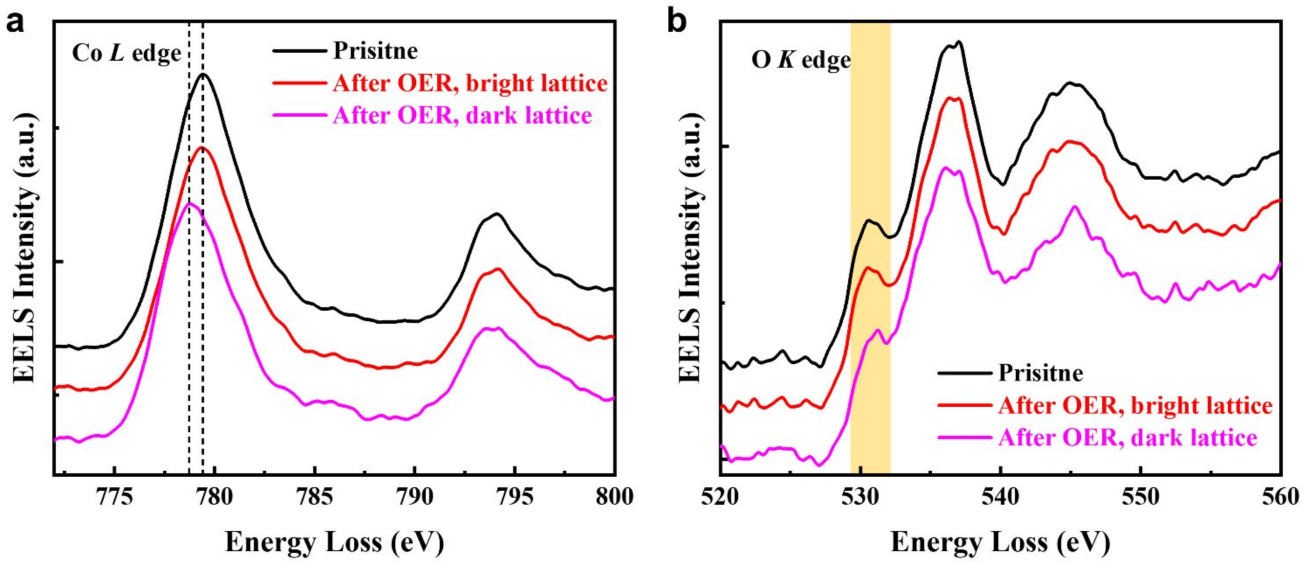

Figure S10 STEM-EELS of LCO films before and after OER. (a) Co-*L* edge and (b) O-*K* edge EELS for LCO/STO film before and after OER.

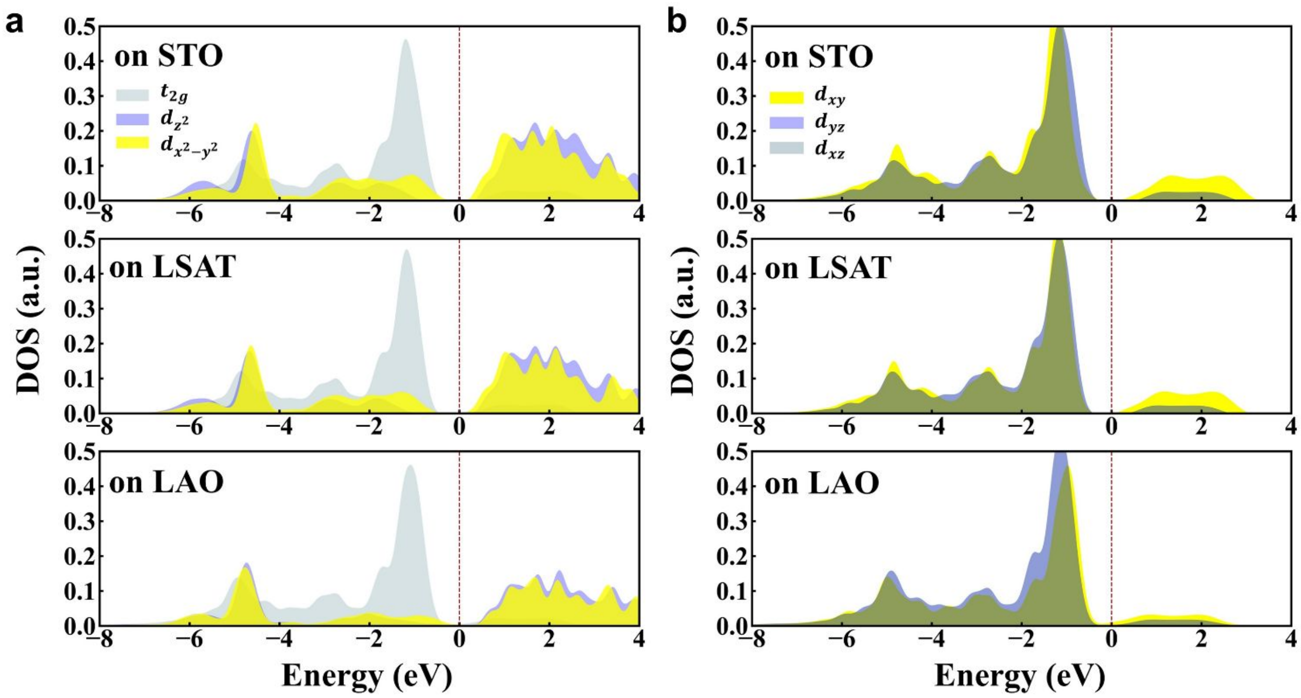

Figure S11 DOS calculations for Co 3*d* in LCO films. Projected density of states (DOS) of (a) e_g and (b) t_{2g} for Co 3*d* orbitals in LCO films, respectively.

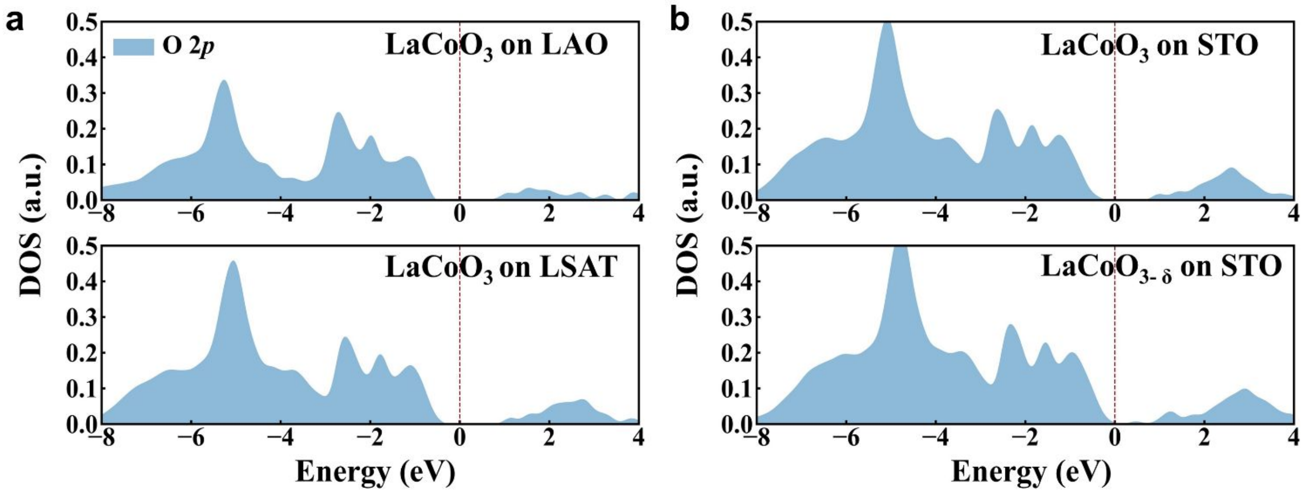

Figure S12 DOS calculations for O 2*p*. (a) Projected density of states (DOS) of O 2*p* orbitals in $LaCoO_3$ films grown on LAO and LSAT, respectively. (b) DOS of O 2*p* orbitals in $LaCoO_3$ and $LaCoO_{3-\delta}$ films grown on STO, respectively.

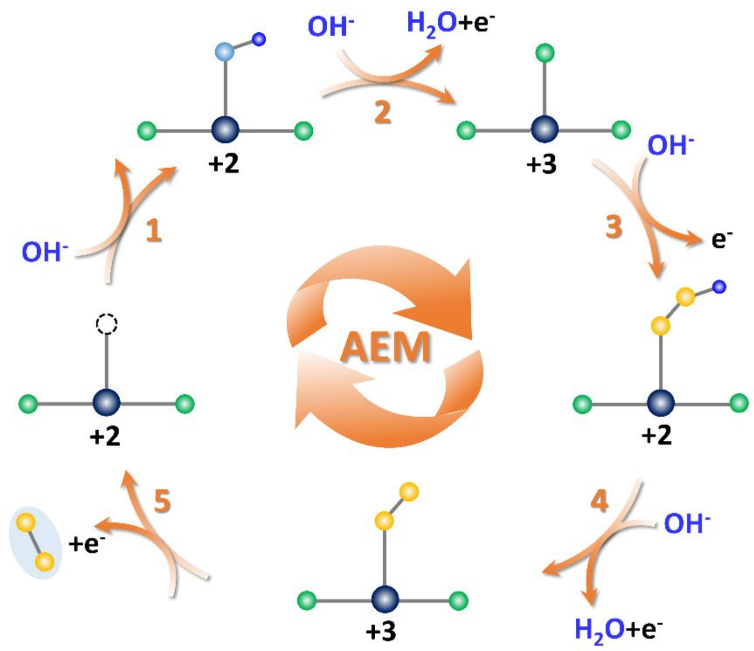

$$4OH^- \rightarrow O_2 + 2H_2O + 4e^-$$

Figure S13 Schematic diagram of AEM pathway for $LaCoO_{3-\delta}$ films

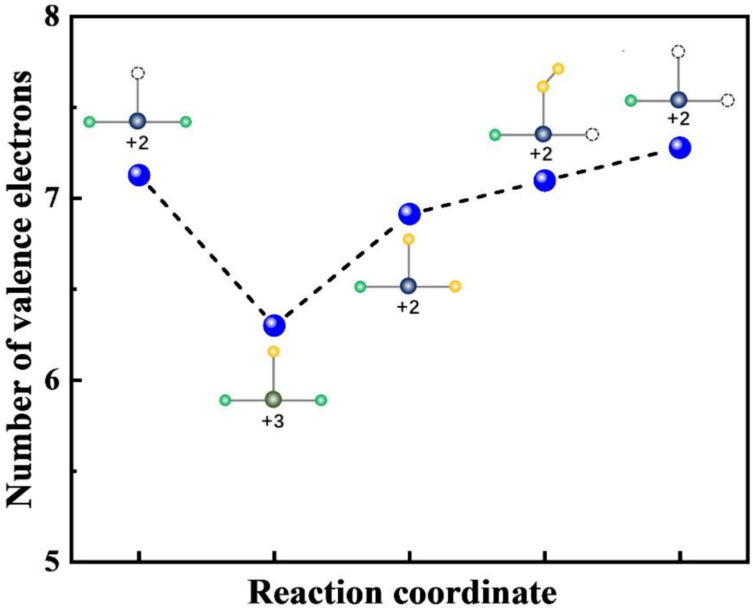

Figure S14 Calculation of valence electron number. Variation of valence electron number of cobalt ion in $LaCoO_{3-\delta}$ films during LOM-like OER.

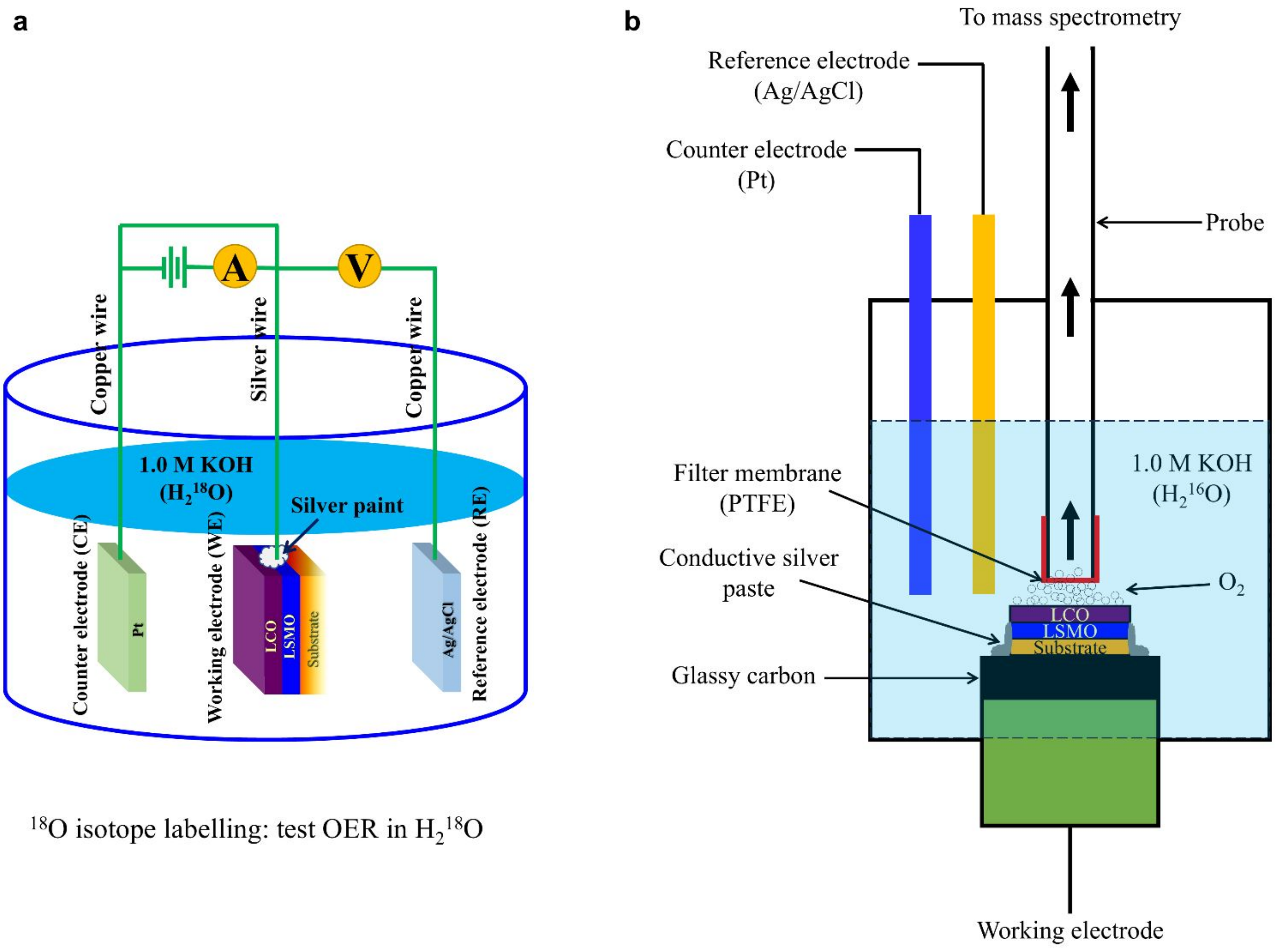

^{18}O isotope labelling: test OER in $H_2{}^{18}O$

The ^{18}O-labeled LCO film test DEMS in $H_2{}^{16}O$

Figure S15 Schematic diagram of the device for the ^{18}O isotope labelling and the DEMS testing system. (a) OER testing in $H_2{}^{18}O$. (b) DEMS testing for the OER catalyzed by the ^{18}O-labeled $LaCoO_3$ films in $H_2{}^{16}O$.

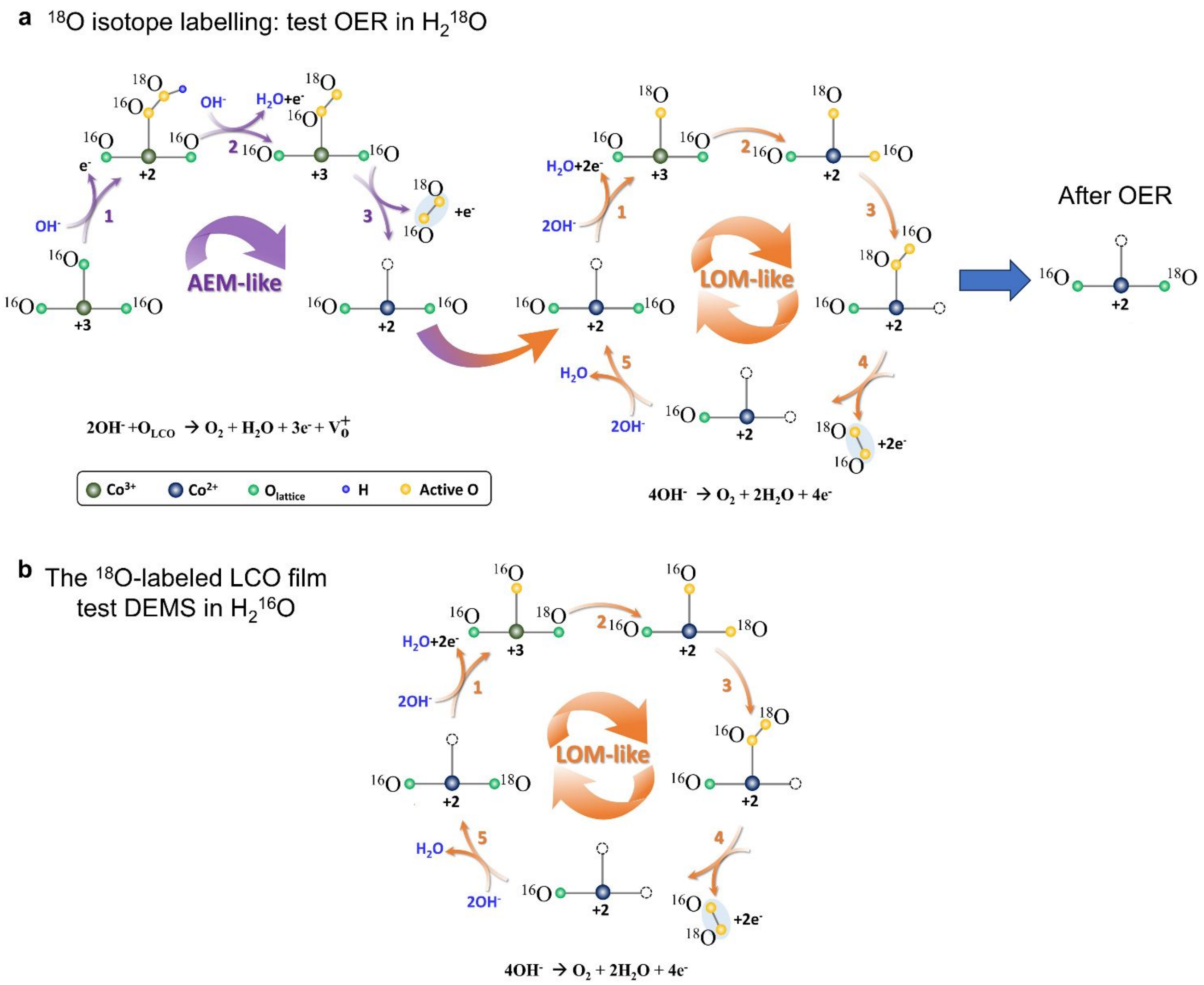

Figure S16 OER pathways on $LaCoO_3$ thin films. (a) isotope ^{18}O labelling via OER in $H_2{}^{18}O$-based electrolyte; (b) $^{34}O_2$ evolution in OER measurement for the ^{18}O-labeled LCO films.
